# Characterization of defense responses against bacterial pathogens in duckweeds lacking EDS1

**DOI:** 10.1111/nph.18453

**Published:** 2022-09-25

**Authors:** Erin L. Baggs, Meije B. Tiersma, Brad W. Abramson, Todd P. Michael, Ksenia V. Krasileva

**Affiliations:** ^1^ Department of Plant and Microbial Biology University of California Berkeley Berkeley CA 94720 USA; ^2^ Plant Molecular and Cellular Biology Laboratory The Salk Institute for Biological Studies La Jolla CA 92037 USA

**Keywords:** antimicrobial proteins, duckweed, EDS1, plant immunity, *Pseudomonas syringae*, transcriptional response

## Abstract

- *ENHANCED DISEASE SUSCEPTIBILITY 1 (EDS1)* mediates the induction of defense responses against pathogens in most angiosperms. However, it has recently been shown that a few species have lost *EDS1*. It is unknown how defense against disease unfolds and evolves in the absence of *EDS1*. We utilize duckweeds; a collection of aquatic species that lack *EDS1*, to investigate this question.
- We established duckweed‐*Pseudomonas* pathosystems and used growth curves and microscopy to characterize pathogen‐induced responses. Through comparative genomics and transcriptomics, we show that the copy number of infection‐associated genes and the infection‐induced transcriptional responses of duckweeds differ from other model species.
- Pathogen defense in duckweeds has evolved along different trajectories than in other plants, including genomic and transcriptional reprogramming. Specifically, the miAMP1 domain‐containing proteins, which are absent in Arabidopsis, showed pathogen responsive upregulation in duckweeds. Despite such divergence between Arabidopsis and duckweed species, we found conservation of upregulation of certain genes and the role of hormones in response to disease.
- Our work highlights the importance of expanding the pool of model species to study defense responses that have evolved in the plant kingdom independent of *EDS1*.

## Introduction

Receptors and signaling components of the plant immune signal network likely evolved before the divergence of flowering plants 181 million years ago (Ma; Kumar *et al*., 2017). The two primary layers of plant innate immunity, microbe‐associated molecular pattern (MAMP)‐triggered immunity (MTI) and effector‐triggered Immunity (ETI; Alhoraibi *et al*., 2019), form an intricate network of cross‐amplifying pathways (Ngou *et al*., 2021; Tian *et al*., 2021; Yuan *et al*., 2021a). The shared evolutionary history of MTI and ETI suggests that they intricately co‐evolved and are foundational for immunity in flowering plants. ENHANCED DISEASE SUSCEPTIBILITY 1 (EDS1) is a central hub in MTI and ETI signal transduction and amplification. Mutations abolishing activity of EDS1 compromise ETI and MTI (Wagner *et al*., 2013; Cui *et al*., 2017; Pruitt *et al*., 2021). Surprisingly, genomic studies have revealed several angiosperms that thrive in their natural environments without EDS1, including the Lemnaceae (Baggs *et al*., 2020). It is unknown how the response to bacterial pathogens would proceed or evolve in the absence of a functional EDS1.

Lemnaceae diverged from other monocots at 128 Ma (Kumar *et al*., 2017). The common ancestor of the Lemnaceae was present in a freshwater aquatic environment and had a reduced body plan of a frond (a fused stem and leaf) and root (Acosta *et al*., 2021). Genera within the Lemnaceae include *Spirodela*, *Landoltia*, *Lemna*, *Wolffia* and *Wolffiella*, all of which primarily reproduce through asexual budding (Bog *et al*., 2019). The small genome and body plan is conducive to the Lemnaceae's rapid lifecycle with a doubling time of as little as 34 h (Michael *et al*., 2020). Lemnaceae are very easy to grow in the laboratory which has stimulated research into their use as biofuels (Su *et al*., 2014; Van Hoeck *et al*., 2015; Xu *et al*., 2018) and enabled the growth of genomic resources (Wang *et al*., 2014; Michael *et al*., 2017, 2020; Hoang *et al*., 2020; Abramson *et al*., 2021). *Spirodela polyrhiza* has a small genome of 158 Mb and has lost members of the expansin and cellulose biosynthesis families (Wang *et al*., 2014; Michael *et al*., 2017). *Wolffia australiana* has lost light signaling pathways and root development pathways, which is not surprising given the absence of roots in *Wolffia* (Michael *et al*., 2020). In addition to developmental pathways lost in other Lemnaceae, *S. polyrhiza* was previously shown to have lost *EDS1* (Baggs *et al*., 2020), however the extent of immune pathway loss in other Lemnaceae remained unclear.

Given the absence of the EDS1 pathway in *S. polyrhiza* (Baggs *et al*., 2020), we hypothesized that duckweeds could be a model system for understanding EDS1‐independent innate immunity. Lemnaceae are globally distributed and only absent from the Arctic and Antarctica (Landolt, 1992; Crawford *et al*., 2006); several Lemnaceae species are invasive species (Abramson *et al*., 2021; CABI, 2021). The wide distribution of duckweeds means their range overlaps with several model plant pathogens: *Pseudomonas syringae* pv *tomato* DC3000, *P. syringae* pv *syringae*, *Xanthomonas perforans*, *Xanthomonas euvesicatoria* (Cai *et al*., 2011; Potnis *et al*., 2015; Gutiérrez‐Barranquero *et al*., 2019), and many others for which genomic and genetic resources are available. These pathogens often have devastating effects on crop plants yield and value (Martins *et al*., 2018).

Microbe‐associated molecular pattern‐triggered immunity immune signaling is triggered by the recognition of conserved molecular patterns through pattern recognition receptors (PRRs) and their respective co‐receptors. There are two main types of PRRs: receptor‐like kinases (RLKs) and receptor‐like proteins (RLPs), that lack an intracellular kinase domain (Lolle *et al*., 2020; Offor *et al*., 2020; Yuan *et al*., 2021b). Substantial differences exist between RLP and RLK signaling pathways: immune signaling through the RLP and Suppressor of BIR1 (SOBIR1) co‐receptor pathway genetically requires *EDS1*, *Phytoalexin Deficient 4* (*PAD4*) and *Resistance to Powdery Mildew 8 Nucleotide‐binding Leucine‐rich repeat Receptors* (*RNLs*) and results in higher levels of ethylene and phytoalexins production, as well as Pathogenesis Related 1 gene expression (Pruitt *et al*., 2021; Tian *et al*., 2021). *Pathogenesis Related* (*PR*) genes are characterized by their rapid upregulation after pathogen infection, they include several antimicrobial genes with different modes of action (glucanase, thaumatin, chitinase, thionin and defensin; Ali *et al*., 2018). There are many antimicrobial peptides that are not classed as *PR* genes, such as proteins with the MiAMP1 domain (Marcus *et al*., 1997; McManus *et al*., 1999; Stephens *et al*., 2005). The MiAMP1 domain was identified as a key structural motif in the MiAMP1 protein named after its isolation from *Macadamia integrifolia* and its antimicrobial activity (Marcus *et al*., 1997; Stephens *et al*., 2005). Furthermore, transgenic canola expressing MiAMP1 showed enhanced resistance to the pathogen *Leptosphaeria maculans* (Kazan *et al*., 2002). The diversity and modes of action of these antimicrobials remains poorly understood.

Receptor‐like protein and RLK activation primes the cell to initiate a stronger immune response upon Nucleotide‐binding Leucine‐rich repeat Receptors (NLRs) perception of intracellular changes caused by pathogen‐derived effectors (Tian *et al*., 2021). Conserved domain architecture and signaling specificities of NB‐ARC domain‐containing proteins allow their classification into RNLs, coiled‐coil NLRs (CNLs), Toll/interleukin‐1 receptor NLRs (TNLs) and TIR‐NBARC‐Tetratricopeptide repeats (TNP; Nandety *et al*., 2013; Shao *et al*., 2016, 2019; Baggs *et al*., 2017; Johanndrees *et al*., 2021). Disease resistance mediated by TNLs and some CNLs is genetically dependent on RNLs and the lipase‐like proteins EDS1 and PAD4 or SAG101. The recognition of pathogen presence by an NLR typically leads to qualitative resistance where the plant shows a discrete resistant phenotype.

In the absence of NLR triggered ETI, plants are not necessarily susceptible to a pathogen. Instead, quantitative resistance may be observed where extent of resistance is more variable and, in some cases, underpinned by hundreds of loci (Corwin & Kliebenstein, 2017). Mechanisms implicated in quantitative resistance include defensins, pathogenesis‐related proteins, secondary metabolite enzymes and pathogen‐induced phytohormones accumulation (Corwin & Kliebenstein, 2017). Qualitative disease resistance follows mendelian patterns of inheritance of resistance of individuals to a given pathogen genotype. Typically, the mechanism of qualitative resistance involves MTI and ETI. In contrast, the level of quantitative resistance spans a continuous distribution and is typically governed by several small effect loci.

In this study, we investigated the phenotypic, genomic, and transcriptomic characteristics of duckweeds in response to *Pseudomonas* phytopathogens. We found a stepwise reduction of conserved ETI pathways within the Lemnaceae and loss of MTI immune pathway components in *W. australiana*. However, we observed a shared expansion of the MiAMP1 protein family across Lemnaceae. Additionally, we noticed species‐specific responses to pathogen treatments among duckweed species. We investigated the transcriptional response to pathogens of duckweed species, a system which is adapted to the absence of EDS1‐mediated immune signaling cascades. Our study highlights duckweeds as a rapid growth, high throughput, minimalist MTI‐ETI model organism. As such, it could be utilized to expedite our understanding of EDS1‐independent MTI‐ETI mechanisms of immunity as well as to dissect mechanisms of quantitative resistance.

## Materials and Methods

### *Landoltia punctata* clone 5635 DNA isolation and sequencing

*Landoltia punctata* clone 5635 (Lp5635, formerly DWC138) was received from the Rutgers Duckweed Stock Collective (RDSC; http://www.ruduckweed.org/). Lp5635 was collected, patted dry to remove excess water and flash frozen in liquid nitrogen. Frozen tissue was ground with a mortar and pestle in liquid nitrogen. High molecular weight (HMW) DNA was isolated using a modified Bomb protocol (Oberacker *et al*., 2019). DNA quality was assessed on a Bioanalyzer and HMW status was confirmed on an agarose gel. Libraries were prepared from HMW DNA using NEBnext (NEB, Beverly, MA, USA) and 2 × 150 bp paired end reads were generated on the Illumina NovaSeq (San Diego, CA, USA). Resulting raw sequence was only trimmed for adaptors, resulting > 60× coverage of the diploid *L. punctata* genome (350 Mb).

### *Landoltia punctata* clone 5635 genome assembly, gene prediction and annotation

Illumina paired end reads were assembled with Spades (v.3.14.0) with the default settings (Bankevich *et al*., 2012). Resulting contigs were annotated using a pipeline consisting of four major steps: repeat masking, transcript assembly, gene model prediction, and functional annotation as described (Abramson *et al*., 2021). Repeats were identified using Edta (v.1.9.8; Ou *et al*., 2019) and these repeats were used for softmasking. RNA‐sequencing (RNA‐Seq) reads were aligned to the genomes using Minimap2 and assembled into transcript models using Stringtie (v.1.3.6). The soft‐masked genome and Stringtie models were then processed by Funannotate (v.1.6; https://github.com/nextgenusfs/funannotate) to produce gene models. Predicted proteins were then functionally annotated using Eggnog‐mapper (v.2; Huerta‐Cepas *et al*., 2017).

### Pathway ortholog identification

The genomes and proteomes used included *Arabidopsis thaliana* TAIR 10 (Lamesch *et al*., 2012), *Solanum lycopersicum* ITAG2.3 (Tomato Genome Consortium, 2012), *Oryza sativa* v.7 (Ouyang *et al*., 2007), *S. polyrhiza* v.2 (Wang *et al*., 2014), *W. australiana* 8730 (Michael *et al*., 2020), *Colocasia esculenta* (L.) Schott (*C. esculenta* S; Yin *et al*., 2021) and *C. esculenta* Niue (*C. esculenta* N) (Atibalentja *et al*., 2019). Proteome completeness was evaluated using Busco v.5 (Simão *et al*., 2015; Table S1).

To date the loss of *EDS1*, *PAD4*, *ADR1* and *NDR1*, we ran a Blastp search using the *O. sativa* and *A. thaliana* EDS1, PAD4, ADR1 and NDR1 proteins as queries against two Taro proteomes; *C. esculenta* (L.) Schott (*C. esculenta* S; Yin *et al*., 2021) and *C. esculenta* N. If no ortholog was identified, we used tBlastn to check this was not an artifact of annotation. To confirm orthologs, the Taro subject sequence was the query for Blastp to the *O. sativa* and *A. thaliana* proteomes. Upon confirming the presence of *EDS1*, *PAD4*, *ADR1* and *NDR1* in Taro, we utilized TimeTree.org to estimate the time during which *EDS1*, *PAD4*, *ADR1* and *NDR1* were lost.

To identify orthologous genes, Orthofinder (v.2.5.4; Emms & Kelly, 2019) was ran on *S. polyrhiza* 7498 (Wang *et al*., 2014), *L. punctata* 5635, *W. australiana* 8730 (Michael *et al*., 2020) and *A. thaliana* TAIR 10 (Lamesch *et al*., 2012). Infection responsive *A. thaliana* genes were identified by literature searches (Table S2). The Arabidopsis GIDs were used to extract orthogroups containing Lemnaceae homologs which were cross checked using Phytozome. Gene absence was verified using tBlastn (Camacho *et al*., 2009). For large gene family analysis of MiAMP1, RLK, RLP and NLRs, pfamscan (Sarris *et al*., 2016; Madeira *et al*., 2019) was used to identify proteins with domains of interest (*e*‐value = 10; MiAMP1, RLK, RLP and NB‐ARC domains; Table S1). Receptor‐like kinase, RLP and MiAMP1 protein sequences were aligned with Muscle v.3.8.1551 (Madeira *et al*., 2019) and NB‐ARC domains were aligned with hmmalign from hmmer3 (Wheeler & Eddy, 2013) to NB‐ARC1‐ARC2 (Bailey *et al*., 2018). Alignments were manually curated using Belvu (Barson & Griffiths, 2016) and Jalview (Waterhouse *et al*., 2009). Maximum likelihood phylogenies were calculated utilizing RAxML (v.8.2.9; ‐f a, ‐x 12 345, ‐p 12345, ‐# 100, ‐m PROTCATJTT).

### Plant growth conditions

Duckweed fronds were propagated by transferring three mother fronds to a new well containing fresh media every 3–4 wk. Plants were grown on Schenk and Hildebrandt basal salt media (S6765‐10L, 0.8% agarose, pH 6.5; Sigma‐Aldrich) in six‐well plates and then placed in a growth chamber set to 23°C with a diurnal cycle of 16 h : 8 h, light (75 μmol) : dark.

### Pathogen inoculation

Bacterial colonies were grown in Luria Broth (LB) media supplemented with appropriate antibiotics (10 μg ml^−1^; kanamycin (Km), 50 μg ml^−1^; rifampicin (Rif), 50 μg ml^−1^; spectinomycin (Sp)) overnight at 28°C in a shaking incubator (20.5 g). Pathogens used in this study included: *P. syringae* pv *tomato* DC3000 GFP (Matthysse *et al*., 1996; Mudgett & Staskawicz, 1999), *P. syringae* pv *tomato* DC3000 *cma* (Sreedharan *et al*., 2006), *P. syringae* pv *tomato* DC3000 *hrcC* (Mudgett & Staskawicz, 1999), *P. fluorescens* N2C3 (DSM 106121; Parte *et al*., 2020), *P. syringae* B7281 (Feil *et al*., 2005), *P. syringae* pv *glycinea* race 4 (Staskawicz *et al*., 1987), *P. syringae* pv *tabaci* 11528 (Institute of Medicine (US) Committee on Resource Sharing *et al*., 1996), *P. syringae* pv *maculicola* (Davis *et al*., 1991), *X. euvesicatoria* (Roden *et al*., 2004), *X. gardneri* (Schwartz *et al*., 2017), *X. perforans* (Bophela *et al*., 2019), and *X. translucens* (Peng *et al*., 2016). All strains were plated on Rif; *P. syringae* pv *tomato* DC3000 on Rif/Km and *P. syringae* pv *tomato* DC3000 *cma* on Rif/Km/Sp. Liquid culture was centrifuged (3000 ***g***, 15 min). The pellet was resuspended in 10 mM magnesium chloride (MgCl_2_), the optical density measured at a wavelength of 600 nm (OD_600_) was determined, and the infiltration solution was diluted to a final standard high inoculum of OD_600_ = 0.1, equivalent to 1 × 10^8^ cells of *Pst* DC3000. Then 500 μl of OD_600_ = 0.1 solution was pipetted on to three duckweed fronds per well. The plate was then returned to the incubator or placed in vacuum (0.8 PSI) for 10 min.

For growth curves, bacterial cells were resuspended at 1 × 10^8^ colony‐forming unit (CFU) ml^−1^, OD_600_ = 0.1 in 10 mM MgCl_2_. The inoculum was diluted to a standard low inoculum with a final concentration of 1 × 10^5^ CFU ml^−1^. Each well was inoculated with 500 μl of treatment, followed by vacuum infiltration (0.8 PSI) for 10 min. For each timepoint 1 cm^2^ of fronds was sampled in 150 μl MgCl_2_ and glass beads, then homogenized by a biospec mini‐beadbeater (2000 rpm, vital distance 3.175 cm). Serial dilutions were made and plated on selective media. Two days after plating, colonies were counted.

### Microscopy

For microscopy of duckweed treated with bacteria, flood inoculation with 500 μl solutions OD_600_ = 0.1 were used. Whole fronds were staged in water on slides and covered with a glass coverslip. The slides were imaged on a Zeiss 710 LSM confocal microscope (Zeiss) with either the 20× (water), 63× (oil) or 100× (oil) objectives. To image bacteria on duckweed fronds, they were stained with 10 μl of 1× Syto™ BC Green Fluorescent Nucleic Acid Stain (S34855; Thermo Scientific, Waltham, MA, USA). Duckweed fronds treated with SA were imaged on an Olympus SZX12 stereo microscope.

### Hormone supplementation

Coronatine (C8115‐1MG; Sigma‐Aldrich) powder was dissolved in 100% dimethyl sulfoxide (DMSO) to create a 200 μM ml^−1^ stock that was then diluted to 3 and 0.3 μM in double‐distilled water (ddH_2_O). Salicylic acid (SA) BioXtra ≥ 99.0% (S5922‐100G; Sigma‐Aldrich) was diluted in water to concentrations of 2, 0.4 and 0.2 mM. Buffer solution used was the same as the solvent for the treatment. Individual wells of 6 or 12 well plates containing three duckweed fronds were inoculated with 500 or 250 μl of phytohormone solution, respectively. Phytohormone treatments were applied just before pathogen treatment.

### RNA‐Seq analysis

Inoculations were conducted as outlined earlier using standard high bacterial inoculum 1 × 10^8^ CFU ml^−1^. At 30 min, 1 h, 6 h and 12 h after inoculation, fronds from the same well were transferred to tubes containing 1.5 ml RNA later and frozen in liquid nitrogen. RNA was extracted using Qiagen RNAeasy plant kit (74903; Qiagen) and samples with a RNA integrity number score of > 8.0 (Agilent Bioanalyzer 2100 performed by QB3‐UC Berkeley, Berkeley, CA, USA) were used for library preparation. Library construction and sequencing were performed by Novogene (Sacramento, CA, USA) using NEBNext Ultra II RNA library prep by Illumina (E7770; Illumina, San Diego, CA, USA) and an Illumina Novaseq 6000 S4. After sequencing, reads were demultiplexed using Illumina indices and a quality control (QC) check removed ‘N’ containing, low quality, and adapter‐related reads. Upon receiving the data from Novogene, an initial QC of reads was performed with Fastqc (Andrews, 2010). Reads were then mapped to the *S. polyrhiza* v.2 genome using Hisat2 (Kim *et al*., 2015). Read coverage tables were computed using Stringtie (Pertea *et al*., 2015) and differential gene expression analysis was carried out using edgeR (Dai *et al*., 2014). Genes were considered differentially expressed if they met the criteria ¦log_2_FC¦ > 1, FDR < 0.05 (FC, fold change; FDR, false discovery rate). For a detailed list of commands see: https://github.com/erin‐baggs/DuckweedRNA. Outliers were removed by visual inspection; the edgeR count tables were plotted as PlotMDS method log_2_FC and BCV (Figs S1–S4). Samples were removed if they alone were causing most of the variance on a dimension leading to all other samples clustered into a corner. Then, if one sample of a treatment was grouping with samples of an opposing treatment, gene expression was analyzed to check whether the sample's expression pattern may have been the result of cross contamination. If the expression was inconsistent with at least three other replicates from the treatment group it belonged to, the sample was removed. Replicates removed from *S. polyrhiza* analysis included B3, B20, B19, P21, B34, P114. The replicates B15, B20, P39, P70, H80, B94, P103 and H115 were removed from *L. punctata* analysis.

Orthofinder (Emms & Kelly, 2019) was used to identify orthogroups between *A. thaliana* and duckweeds. *Arabidopsis thaliana* gene identifiers of marker genes were then used to extract the gene identifiers of duckweed homologs. The *S. polyrhiza* and *A. thaliana* homologous relationships were then cross‐referenced by comparing to Phytozome protein homologs. Phytozome was then used to infer *O. sativa* homologs. The differential expression of the duckweed homologs to *A. thaliana* marker genes was extracted from edgeR data and plotted in R genevestigator (https://genevestigator.com; Hruz *et al*., 2008) was used to identify the differential expression of *A. thaliana* homologs to duckweed upregulated genes in two public Affymetrix Arabidopsis ATH1 genome array (AtGenExpress: ME00331 (Kemmerling *et al*., 2011) and GEO: GSE5520 (Thilmony *et al*., 2006)) a third array was used to investigate ABA response (Array Express: E‐MEXP‐2378 (Umezawa *et al*., 2010)). The differential expression *O. sativa* homologs to duckweed conserved pathogen upregulated genes was extracted from the EBI Expression atlas (Athar *et al*., 2019).

## Results

### Lemnaceae condensed many immune signaling components and expanded the MiAMP1 protein family

**Fig. 1:**
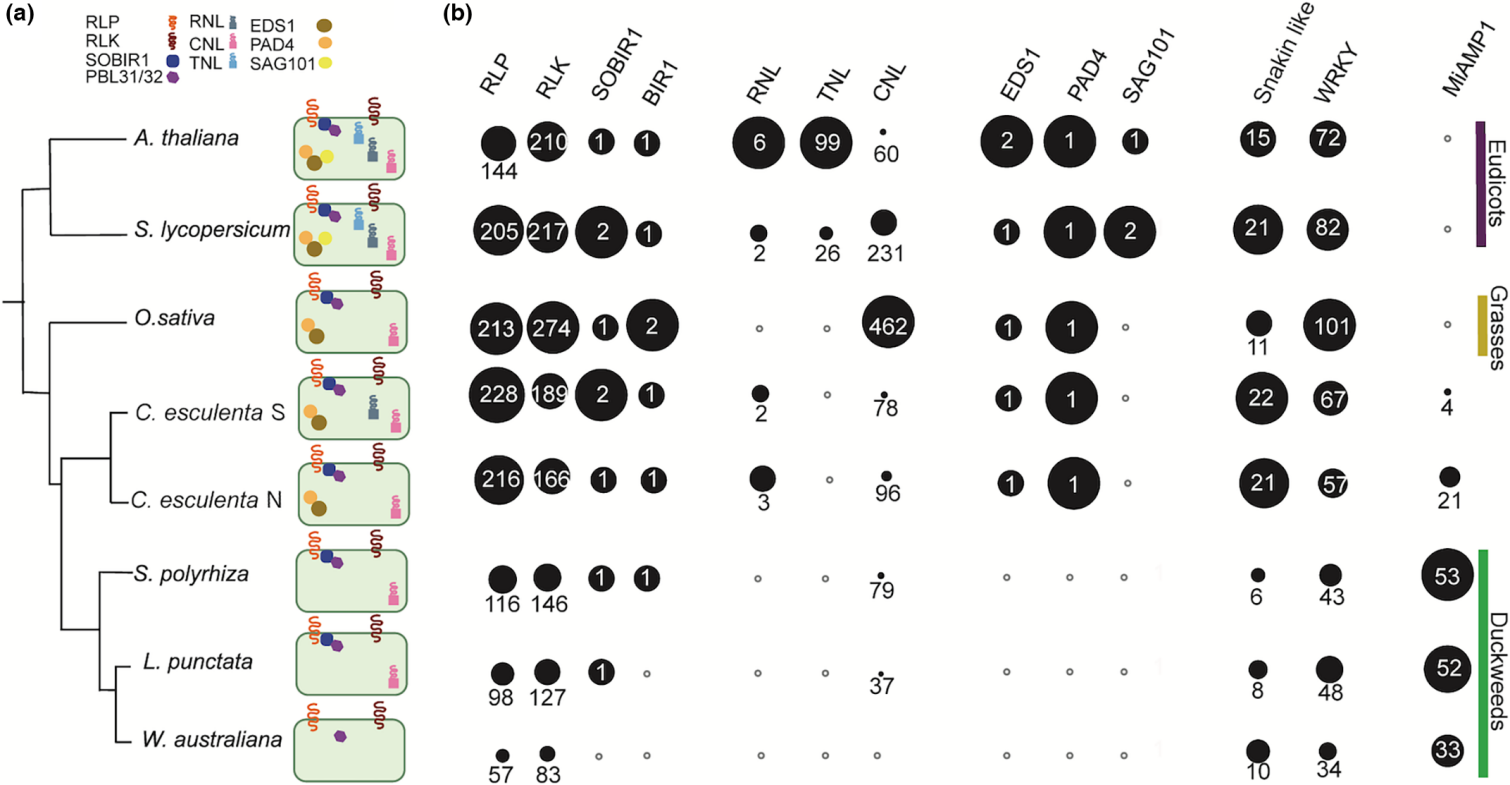
Copy number variation of conserved angiosperm immune signaling components. (a) Phylogenetic relationship of duckweeds to other representative angiosperms and depiction of presence of signaling components. If components were not identified by Orthofinder, reciprocal Blastp, and tBlastn, the representative symbol was not displayed in the cell. (b) The size of circles within each column are proportional to the highest copy number of that gene family in a given species, the copy number is denoted in white text. White circles with a gray outline indicate no members of that gene family were identified.

To understand how immune genes have diverged within the Lemnaceae, we utilized publicly available genomes of *S. polyrhiza* (Wang *et al*., 2014) and *W. australiana* (Michael *et al*., 2020). Additionally, we assembled and annotated the genome of *L. punctata* clone 5635 (Table S1), which allowed us to catalog the presence and absence variation across three duckweed genera (Fig. 1a; Table S3). Consistent with previous findings *EDS1*, *PAD4*, and *RNLs* were absent across the Lemnaceae (Lapin *et al*., 2019; Baggs *et al*., 2020; Michael *et al*., 2020). To estimate the timing of the EDS1 pathway loss, we used the closest related species to duckweeds with an available genome, giant Taro (*C. esculenta*; Yin *et al*., 2021). Taro has the *EDS1* pathway (Fig. 1a), suggesting that the loss of these genes in Lemnaceae occurred after their divergence from Taro 104–117 Ma (Kumar *et al*., 2017). The identification of several MiAMP1 genes in Taro (Fig. 1b) suggests the expansion of MiAMP1 proteins can occur independently of the loss of EDS1. Without greater depth of sampling of the Araceae family and sister lineages it remains unclear if MiAMP1 expansions are independent or a single expansion event.

To understand the extent of the immune signaling pathways divergence across Lemnaceae, we surveyed the presence of 24 protein families that have a known role in plant disease defense and pre‐date the monocot and dicot divergence. All 24 protein families selected were present in *S. polyrhiza* (Tables S3, S4; Figs S5–S7). Though gene families associated with immunity were often retained in the Lemnaceae, we noticed a reduction in copy number, like NLRs (Figs 1b, S7; Table S3). Only three NB‐ARC domain‐containing proteins are present in *W. australiana* (Michael *et al*., 2020): two of contain only an NB‐ARC and the other is a TNP whose conservation is consistent with TNPs being EDS1 independent (Johanndrees *et al*., 2021). *Wolffia australiana* has retained many other immune components. While we identified no orthologs of *Pathogenesis related* 4 (*PR4*), *SOBIR1* and *BAK1 interacting receptor 1* (*BIR1*) in *W. australiana*, they were present in *L. punctata* and *S. polyrhiza* (Fig. 1b; Table S3). Our observations indicate that immune pathways have undergone varying degrees of gene loss across the duckweed genera.

Despite the trend towards reductionism of immune signaling components in the Lemnaceae, we identified high copy numbers of MiAMP1 domain‐containing proteins. It has previously been shown that antimicrobial proteins in general are expanded in *S. polyrhiza* 7498 (An *et al*., 2019). Phylogenies of Lemnaceae MiAMP1 proteins show lineage specific expansions and rapid birth and death (Fig. S8) suggesting that selection pressures may be favoring their diversification.

### Duckweed species show variable symptoms upon *Pseudomonas* and *Xanthomonas* challenge

**Fig. 2:**
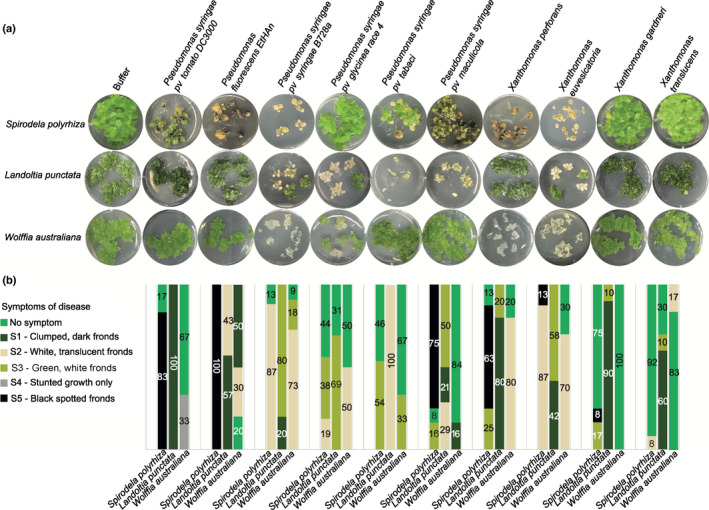
Phenotypic response of duckweed species to bacterial pathogen treatments. (a). One replicate is shown per treatment (full experiment has been independently replicated three times, see Dataset S1; Table S5). Each well displays the most prevalent visual symptom of infection 3 wk after pathogen treatment of five frond clusters. (b) Bar graph showing the percentages of wells displaying each symptom among all replicates (Table S5). Colors of bars correspond to color legend of disease symptoms.

Since Lemnaceae species lack the EDS1 pathway, it was unclear how they would respond to bacterial pathogen infection. We challenged *S. polyrhiza*, *L. punctata*, and *W. australiana* to a panel of common *Pseudomonas* and *Xanthomonas* plant pathogens with a standard high bacterial inoculum (1 × 10^8^ CFU ml^−1^; Fig. 2a). We observed variability among replicates in the susceptibility and resistant phenotypes (Dataset S1; 2b) which is consistent with quantitative resistance. The variability was observed when experiments were started with a single (Fig. S9) or three mother fronds (all other experiments). We identified a few virulent pathogens that produced similar symptoms across all hosts (*X. gardneri*, *P. syringae* pv *tabaci*) while others caused distinct symptoms on a given host species. The most common disease symptoms were chlorosis and reduced growth rate. Surprisingly, despite having fewer NLRs and lacking *SOBIR1* and *BIR1*, the growth of *W. australiana* upon pathogen infection was often less stunted than that of other duckweed species.

For further experiments, we focused on *P. syringae* pv *tomato* DC3000 (*Pst* DC3000) and *P. syringae* pv *syringae* B728a (*Pss* B728a) as there are known virulence factors, large resources of mutants, and the severity of disease symptoms caused varied across duckweed species.

### *Pseudomonas syringae* colonizes the substomatal cavity in *Spirodela polyrhiza* and infection is slowed in T3SS mutants

To characterize the pathology of *Pseudomonas* on duckweed, we used microscopy and bacterial genetics, taking advantage of the type III secretion deficient mutant *Pst* DC3000 *hrcC*.

**Fig. 3:**
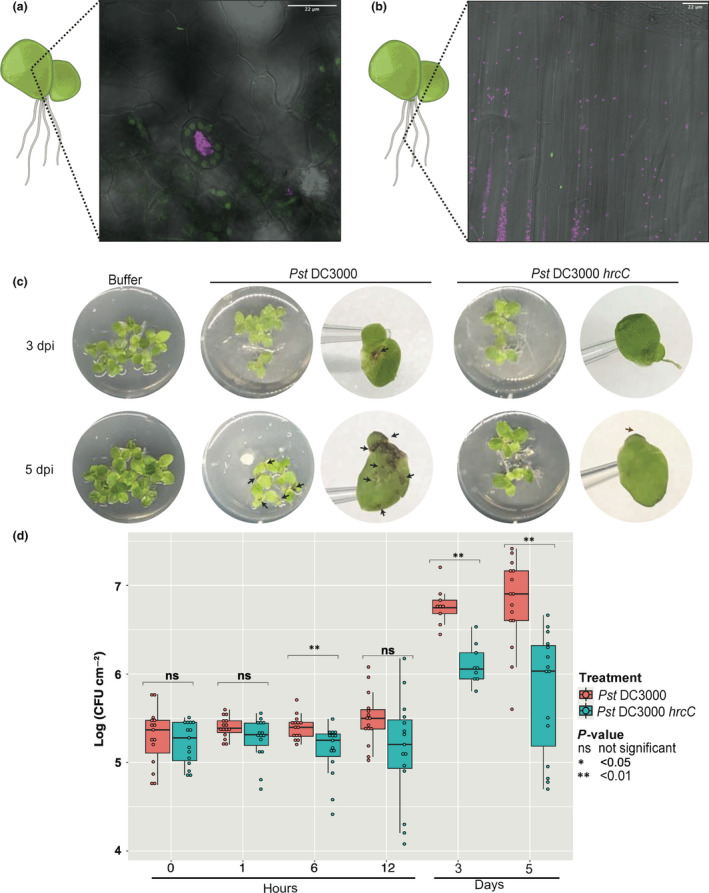
*Pst* DC3000 infection of *Spirodela polyrhiza*. Confocal ×100 microscopy of *Spirodela polyrhiza* inoculated with *Pst* DC3000. False colors: pink – *Pst* DC3000, green – plastids, gray – transmitted light. (a) Frond surface with stomata in the center of frame. (b) Root tissue. (c) Images of duckweed fronds and symptoms at 3 and 5 d post inoculation (dpi) with *Pst* DC3000 and *Pst* DC3000 *hrcC*. The *Pst* DC3000 *hrcC* mutant lacks the type III secretion system used for deployment of effectors to the plant. Individual fronds were photographed on a 10 μl tip to indicate size of fronds. Arrows highlight areas where symptoms are present. (d) Box plot showing the number of colony‐forming units (CFUs) of *Pst* DC3000 and *Pst* DC3000 hrcC at different time points on *Spirodela polyrhiza* fronds. The top horizontal line of each box represents the upper quartile followed by the median and lower quartile. The range extends between the smallest data point within the first quartile value subtract 1.5× the interquartile range and the largest data point within the third quartile value add 1.5× the interquartile range. Colored circles indicate individual data points. Mean‐*t* statistic with Holm adjustment used to assess statistical difference between treatments.

Microscopy of flood inoculated *S. polyrhiza* fronds with standard high bacterial inoculum at 5 d post inoculation (dpi) showed *Pst* DC3000 populations were concentrated at the node (root and frond joint) and budding pockets (where daughter fronds emerge; Fig. S10). By 7 dpi, *Pst* DC3000 populations were observed at the stomata and within the substomatal cavity and mesophyll (Fig. 3a; Dataset S2). We also observed *Pst* DC3000 populations on root tissue at 7 dpi (Fig. 3b). *Pst* DC3000 *hrcC* infection resulted in surface populations on the frond at 5 dpi (Fig. S11a). Individual bacteria were present in the mesophyll (Fig. S11b) but no clear sub‐stomatal populations. At 7 dpi like *Pst* DC3000 the *Pst* DC3000 *hrcC* populations were localized at the frond node (Fig. S11c) however, much smaller areas were colonized. The pattern of small surface populations and individual bacteria in the mesophyll remained the same at 5 and 7 dpi with *Pst* DC3000 *hrcC* (Fig. S11d,e). Together, our observations suggest that the visual symptoms of *Pseudomonas* on duckweed are a result of active bacterial infection. Therefore, the duckweed‐*Pseudomonas* pathosystem constitutes a valuable model for understanding disease progression and EDS1‐independent immune responses.

**Fig. 4:**
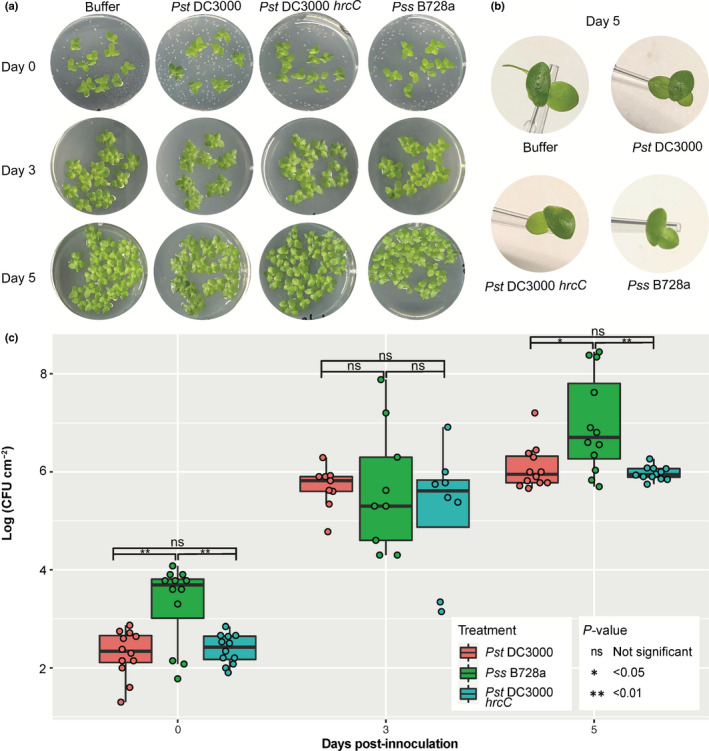
*Pst* DC3000 infection of *Landoltia punctata*. (a) Images of *Landoltia punctata* fronds and symptoms during *Pst* DC3000, *Pst* DC3000 *hrcC* and *Pss* B728a. (b) Individual fronds were photographed on a 10 μl tip to indicate size of fronds. (c) Box plot showing the number of colony‐forming units (CFUs) of *Pst* DC3000, *Pst* DC3000 *hrcC* and *Pss* B728a at different time points on *Landoltia punctata* fronds. The top horizontal line of each box represents the upper quartile followed by the median and lower quartile. The range extends between the smallest data point within the first quartile value subtract 1.5× the interquartile range and the largest data point within the third quartile value add 1.5× the interquartile range. Colored circles indicate individual data points. Mean‐*t* statistic with Holm adjustment used to assess statistical difference between treatments.

To advance our understanding of this pathosystem, we infiltrated *S. polyrhiza* plants with *Pst* DC3000 and *Pst* DC3000 *hrcC* with a low bacterial inoculum (1 × 10^5^ CFU ml^−1^) and monitored bacterial population growth overtime (Fig. 3c,d). The first black lesions were macroscopically visible at 3 dpi with *Pst* DC3000 and by day 5 we observed an increase in the size, number of lesions and proportion of affected fronds (Fig. 3c). Consistent with the dense populations at the budding pocket and node observed by microscopy, macroscopic black lesions observed were initially localized at the budding pocket and node. We observed no black lesions upon inoculation with *Pst* DC3000 *hrcC* at 3 or 5 dpi. In contrast, *L. punctata* infiltrated with *Pst* DC3000 or *Pss* B728a showed no macroscopic lesions even 5 dpi (Fig. 4a,b). Three weeks post inoculation, 5/8 replicates of *L. punctata* inoculated with *Pss* B728a had turned white and produced fewer daughter fronds than in other treatments. However, even at 4 wk post inoculation with *Pst* DC3000 we observed only a slightly darker coloration and clumping. *Pst* DC3000 *hrcC* did not cause any symptoms on *L. punctata* throughout the 3 wk post inoculation (Fig. S12).

*Pst* DC3000 and *Pst* DC3000 *hrcC* were able to proliferate inside *S. polyrhiza* (Fig. 3d). *Pst* DC3000 multiplied to similar levels as previously described in compatible interactions with wild‐type *A. thaliana* (Katagiri *et al*., 2002; Ishiga *et al*., 2011; Velásquez *et al*., 2017). However, we observed significantly fewer colony forming units of *Pst* DC3000 *hrcC* compared to *Pst* DC3000. This suggests that the virulence of *Pst* DC3000 on *S. polyrhiza* relies on the presence of effectors and the type III secretion system. In contrast, consistent with the lack of disease symptoms in *L. punctata* neither *Pst* DC3000 nor *Pst* DC3000 *hrcC* showed significantly different CFU counts at 5 dpi. Instead, both bacterial numbers remained at a level similar to those of *Pst* DC3000 *hrcC* in *S. polyrhiza* (Fig. 4c). Interestingly, although we saw no visible lesions on *L. punctata* 5 d after *Pss* B728a inoculation, the CFU counts were significantly higher than those of *Pst* DC3000 after 5 d. However, the CFU counts of *Pss* B728a in *L. punctata* and *Pst* DC3000 in *S. polyrhiza* were similar. Our results show that *Pseudomonas* pathogens *Pst* DC3000 and *Pss* B728a can proliferate in duckweed host and bacterial multiplication is affected by unknown host factors and by the type III secretion system for *S. polyrhiza*–*P*st DC3000 interaction.

### Hormone treatment effects vary among Lemnaceae spp. and pathogen treatment

**Fig. 5:**
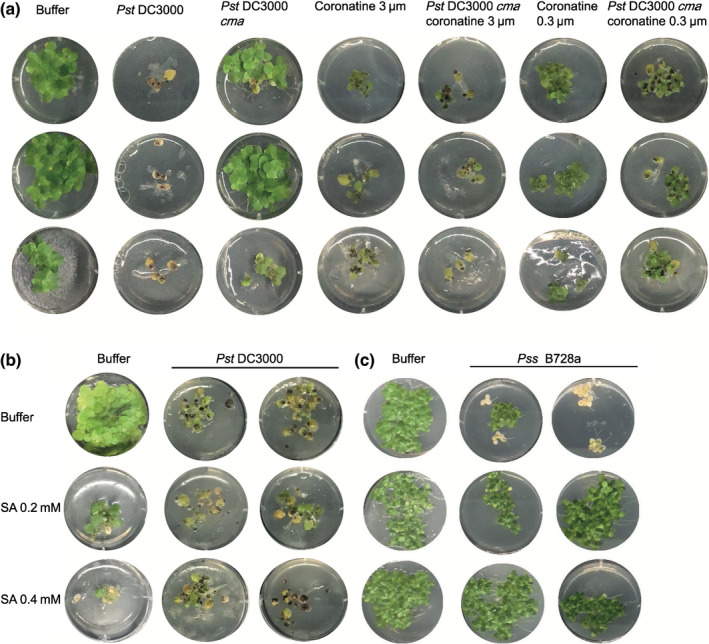
The effects of hormone treatments on *Pst* DC3000 and *Pss* B728a disease symptoms. (a) Images of disease progression 3 wk after the treatment of three *Spirodela polyrhiza* fronds with different concentrations and combinations of coronatine and *Pst* DC3000 mutants (full experiment has been independently replicated three times, see Figs S13, S14). (b) Images of disease progression 3 wk after the treatment of five *S. polyrhiza* fronds with different concentrations and combinations of salicylic acid (SA) and *Pst* DC3000 (full experiment has been independently replicated four times, see Figs S17–S19). (c) Images of disease progression 3 wk after the treatment of five *Landoltia punctata* fronds with different concentrations and combinations of SA and *Pss* B728a treatment, *Pss* B728a was used rather than *Pst* DC3000 as it gives clearer visual symptoms in *L. punctata* (full experiment has been independently replicated three times, see Figs S21, S22).

In response to biotrophy, plants upregulate the phytohormones SA (Cao *et al*., 1994; Clarke *et al*., 1998) and N‐hydroxy pipecolic acid (NHP) through pathways dependent and independent of *EDS1* (Chen *et al*., 2018). To suppress phytohormone responses, *Pst* DC3000 produces the toxin coronatine (Moore *et al*., 1989; Mittal & Davis, 1995), a structural mimic of jasmonic acid (JA) which counteracts SA upregulation (Fonseca *et al*., 2009; Wasternack & Xie, 2010). Since *Pst* DC3000 was virulent on *S. polyrhiza*, we investigated the role of phytohormones and toxins in the absence of *EDS1* through our duckweed pathosystem. The *Pst* DC3000 *cma* mutant is deficient in coronatine biosynthesis and causes mild symptoms on *S. polyrhiza*, marked by the absence of black lesions (Figs 5a, S13, S14). Addition of coronatine alone (0.3 μM) was sufficient to disfigure fronds, reduce growth, and induce the formation of turion resting bodies. The effect on growth and turion formation was stronger at higher concentrations of coronatine (3 μM). However, addition of coronatine on its own was not sufficient to recover symptoms of black lesions and white bleaching of fronds comparable to *Pst* DC3000 infection. The treatment of fronds with coronatine at the time of *Pst* DC3000 *cma* infection restored the black lesions to some extent, but the strain still did not induce white bleaching. Our results show that coronatines role in promoting pathogen virulence is conserved in duckweeds despite the absence of the SA promoting EDS1 pathway.

Given that a core function of the EDS1 pathway is to reinforce SA signaling by inducing several SA responsive genes (Jirage *et al*., 1999; Bonardi *et al*., 2011; Roberts *et al*., 2013; Cui *et al*., 2017); we were interested in how exogenous SA treatment of Lemnaceae would affect disease symptoms. Treatment of Lemnaceae with high levels of SA (2 mM) that are tolerated in *Arabidopsis* were phytotoxic to *S. polyrhiza* (Fig. S15). We therefore carried out experiments using 0.4 and 0.2 mM SA. At these lower concentrations in the absence of a pathogen, we observed dose‐dependent frond disfiguration, growth reduction and an increase in turion formation in *S. polyrhiza*. *L. punctata* only showed growth reduction with 0.4 mM SA (Fig. S16). *S. polyrhiza* co‐treated with SA and *Pst* DC3000 showed typical *Pst* DC3000‐induced symptoms (Figs 5b, S17–S20). However, a small protective effect of SA was observed when *Pst* DC3000 appeared to be less virulent (Fig. S20). We hypothesize that the variation in virulence of *Pst* DC3000 on *S. polyrhiza* affects the ability of SA to restrict pathogen growth below a critical threshold. In the *L. punctata*–*Pss* B728a pathosystem, there was a clear protective effect of SA. Priming of *L. punctata* with SA resulted in loss of the chlorotic symptoms characteristic of the *Pss* B728a treated fronds (Figs 5c, S21, S22). The effect of SA priming on duckweed pathogen interactions appears to be influenced by pathogen strain and plant genotype and complicated by strong endogenous effects of SA on duckweed physiology.

### *Spirodela polyrhiza* and *Landoltia punctata* mount transcriptional responses to *Pst* DC3000

**Fig. 6:**
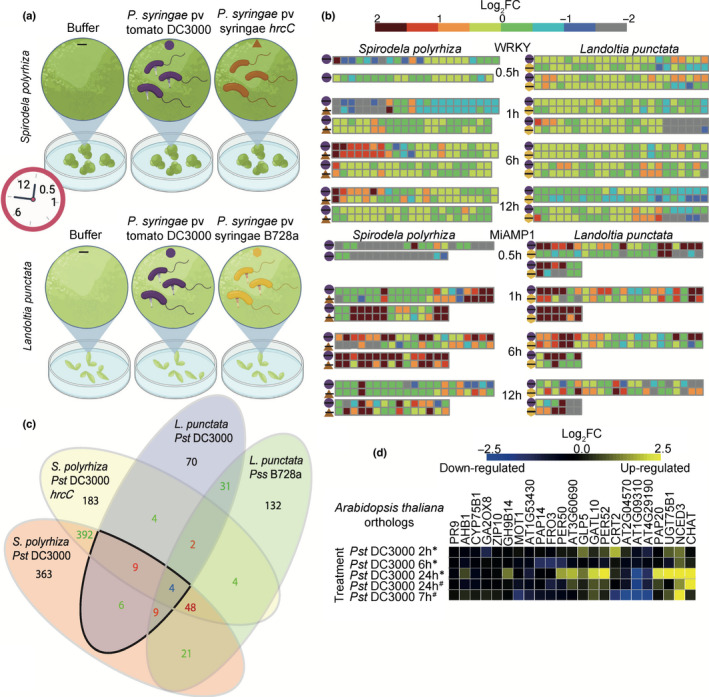
Log_2_‐fold change (FC) of selected gene families following bacterial pathogen exposure of duckweeds. (a) Schematic of the sampling regime for RNA‐sequencing (RNA‐Seq) study. (b) Each square represents a gene with a given domain, the differential expression of the gene is shown by the color of the square. The treatment comparison is indicated by the shapes shown in (a). (c) Venn diagram of numbers of orthogroups that show differential expression (log_2_FC > 1, false discovery rate (FDR < 0.05) in multiple combinations of pathogen treatments and or across different species at any time point. (d) Microarray differential expression analysis of representatives from *Arabidopsis thaliana* Col‐0 of those orthogroups with conserved upregulation upon *Pst* DC3000 in *Spirodela polyrhiza* and *Landoltia punctata*.

Next, we investigated transcriptional response to *Pseudomonas* infection. Despite the absence of *EDS1*, RNA‐Seq revealed a substantial transcriptional response as early as 30 min post infection (Figs 6a, S23; Datasets S3, S4). We first examined gene families that are differentially expressed upon pathogen treatment in other plant species: WRKY (Dong *et al*., 2003), MiAMP1 (Fig. 6b; Adomas & Asiegbu, 2006; Adomas *et al*., 2007), NB‐ARC (Richard *et al*., 2018), and JAZ domain‐containing genes (Ishiga *et al*., 2013; Fig. S24; Tables S6–S13). While we did not observe consistent upregulation log_2_FC > 1 of NB‐ARC and WRKY domain‐containing genes, MiAMP1 domain‐containing genes were consistently upregulated over time in both duckweed species after pathogen treatment.

We hypothesized that there may be some orthologs of *A. thaliana* bacterial‐response genes that are also upregulated in duckweed species upon bacterial infection. Among the 33 *S. polyrhiza* and 32 *L. punctata* genes considered homologous to *A. thaliana* bacterial‐responsive genes (Boudsocq *et al*., 2010; Bjornson *et al*., 2021; Salguero‐Linares *et al*., 2021; Tables S2, S14; Fig. S25), we were able to identify five *S. polyrhiza* and five *L. punctata* gene orthogroups that were significantly upregulated upon pathogen treatment (log_2_FC > 1, FDR < 0.05; Table S15).

Given the suppression of *Pss* B728a symptoms in *L. punctata* upon SA treatment we hypothesized the reduced symptoms seen in *L. punctata* compared to *S. polyrhiza* after *Pst* DC3000 treatment may be due to greater upregulation of SA pathways in *L. punctata*. We investigated the differential expression of duckweed homologs of *A. thaliana* SA biosynthetic and regulated genes (Fig. S26a; Table S16). In *S. polyrhiza* and *L. punctata* we did not observe consistent upregulation of upstream SA biosynthesis genes and regulators (*ICS1*, *NPR1*, *SARD1*, *CBP60g*) or SA catabolism (*DMR6*; Fig. S26b; Tables S17, S18). However, in both duckweed species we saw consistent upregulation of PR1/PR2 ‐like genes which in *A. thaliana* are upregulated downstream of SA production. *L. punctata* also upregulates *WRKY40* homologs after pathogen treatment. Together this indicates that *A. thaliana* and duckweed pathogen transcriptional response pathways converge downstream of SA production and differences in SA signaling is unlikely to explain different disease outcomes between duckweed species.

To investigate which genes have conserved upregulation in *A. thaliana* and duckweeds, we created interspecific orthogroups with Orthofinder (Emms & Kelly, 2019). Then, we identified orthogroups with members that were significantly differentially expressed in both duckweeds after *Pst* DC3000 treatment (Fig. 6c; Table S19; Dataset S5). For orthogroups with conserved upregulation in both duckweeds, we investigated if the *A. thaliana* homologs were similarly upregulated upon *Pst* DC3000 infection, using publicly available datasets (Figs 6d, S27). Among the *A. thaliana* homologs, eight genes showed consistent upregulation after *Pst* DC3000 treatment, including a superoxide dismutase *GERMIN‐LIKE PROTEIN 5*. In contrast upon pathogen treatment, *PATHOGENESIS RELATED 9* (*PR9*) was upregulated in duckweeds but not in *A. thaliana*. There appears to be limited overlap in the transcriptional response to *Pst* DC3000 between *A. thaliana* and duckweeds. Interestingly, half of the orthogroups upregulated in response to bacterial infection, in duckweeds and Arabidopsis were hormone‐regulated or biosynthetic genes suggesting a conserved role of hormones. As the immune pathways of monocots and dicots have diverged substantially, we looked at the differential expression of orthologs of the duckweed upregulated genes in rice across several pathogen treatments (Fig. S28; Table S20). Ten of 28 orthogroups upregulated in duckweeds were also upregulated in rice (log_2_FC > 1.5 across three treatments). Among these genes, five orthogroups included genes also upregulated in *A. thaliana*.

Finally, we identified genes upregulated and unique to duckweeds. Four orthogroups which showed conserved differential expression in both duckweed species do not have homologs in *A. thaliana* (Table S19) of these only two lacked homologs in rice. The two unique orthogroups were cytochrome P450 domain‐containing genes. Cytochrome P450s are present in *A. thaliana* and *O. sativa* (Nelson *et al*., 2004), but lack sequence conservation to those upregulated in duckweeds. MiAMP1 domain‐containing genes are not present in *A. thaliana*. Although MiAMP1 proteins were upregulated across timepoints in both duckweeds, orthogroups containing these proteins did not meet the criteria for conserved upregulation. Since duckweed species do not show a qualitative but a quantitative resistance, we hypothesize that such resistance mechanisms could involve MiAMP1 proteins and cytochrome P450s.

## Discussion

We investigated the immune responses of Lemnaceae whose immune pathways have evolved for *c*. 110 million years in the absence of *EDS1*, a hub for signaling and crosstalk in plant immune system. Whilst the loss of *EDS1* predates the divergence of Lemnaceae, only *W. australiana* has lost the MTI signaling components SOBIR1‐BIR1 (Gao *et al*., 2009; Liebrand *et al*., 2013; Albert *et al*., 2015; van der Burgh *et al*., 2019). Previous work illustrates RLP‐SOBIR1 dependence on EDS1 and PAD4 in *A. thaliana* (Pruitt *et al*., 2021). Despite the loss of *SOBIR1*, compared to other Lemnaceae, *W. australiana* did not have enhanced susceptibility to the bacterial pathogens tested. Given available resources, we began developing and characterizing the pathosystem of *S. polyrhiza* and *L. punctata* interactions with *Pst* DC3000 and *Pss* B728a. Transcriptional and bacterial mutant assays revealed the importance of phytohormones in their response to pathogens; however, differential expression of bacteria responsive *A. thaliana* orthologs was rarely observed. Furthermore, we show that there is a high copy‐number and upregulation upon pathogen infection of MiAMP1 domain‐containing proteins in Lemnaceae.

Duckweeds exist with complex bacterial communities similar to the terrestrial leaf microbiome (Acosta *et al*., 2020). Several duckweed‐associated bacteria have growth promoting effects (O'Brien *et al*., 2020). To understand disease resistance of Lemnaceae to pathogenic bacteria, we screened a range of bacterial pathogens to identify a model pathosystem. Our data shows differences in macroscopic symptoms across Lemnaceae species upon treatment with the same pathogen. Variability was present between fronds within the same treatment well, suggesting there may be environmental and population level factors affecting the outcome of infection. The variability in susceptibility across time points is consistent with quantitative resistance. The invasive nature of duckweeds and numerous healthy populations in the environment raises the questions of how environmental factors may contribute to their quantitative disease resistance. Furthermore, because duckweeds primarily reproduce clonally, have low mutation rates (Sandler *et al*., 2020) and effective recombination (Ho *et al*., 2019), there is limited opportunity for immune proteins to diversify, which is required for generating new resistance specificities. The rather unique combination of duckweeds life‐history traits of fast‐growth, clonal reproduction, reduced morphological complexity and aquatic habitat could influence mechanisms of resistance. In a freshwater habitat, the frequency of contact with MAMPs and microbes likely differs from soil and could affect the balance of the energy trade‐off between growth and defense (Huot *et al*., 2014).

The use of bacterial mutant *Pst* DC3000 *hrcC* revealed the type‐III secretion system can promote virulence of *Pst* DC30000 on *S. polyrhiza*, similar effects have been observed in *A. thaliana* (Hauck *et al*., 2003; Xin *et al*., 2016; Huot *et al*., 2017). Furthermore, our results suggest that virulence of *Pst* DC3000 on *S. polyrhiza* relies on manipulation of host phytohormone pathways through the production of the JA mimic coronatine, similar to in *A. thaliana* (Moore *et al*., 1989; Mittal & Davis, 1995). In contrast, *L. punctata* appears to have a stronger quantitative resistance response to *Pst* DC3000 even in the presence of coronatine. Despite the lack of conservation of the EDS1 pathway in *S. polyrhiza* and *L. punctata*, phytohormones and bacterial toxins remain key mediators of plant–bacterial infection. The reduction of MTI/ETI components in duckweeds opens questions of how duckweeds pathogens evolve; would reduction in effector repertoire be selected due to a reduced pressure for MTI/ETI suppression or would effectors further expand without ETI systems to activate.

Consistent with the variation in phenotypes upon bacterial inoculations, there was a lot of variation that was not explained by pathogen treatment between transcriptomes of duckweed biological replicates. The high variability could be a result of the asynchronous developmental stages of fronds, the quantitative nature of the resistance that we observed within the same population of duckweed or a combination of factors. The use of whole plant tissues likely diluted the signal of differential gene expression. In future studies, single cell transcriptomics might be informative for studying quantitative resistance phenotypes. Despite the variability, our transcriptomic analysis revealed that among the genes consistently differentially expressed after *Pseudomonas* treatment were MiAMP1‐domain‐containing proteins. We hypothesize MiAMP1‐domain proteins maybe upregulated as a nonspecific response to infection, that protects against some but not all pathogens. Such consistent upregulation invites the speculation that they could function against pathogens similarly to MiAMP1 from Macadamia (McManus *et al*., 1999; Stephens *et al*., 2005) which was shown to have anti‐microbial activity against gram‐positive bacteria and fungal pathogens (Marcus *et al*., 1997; Kazan *et al*., 2002; Stephens *et al*., 2005). Thus, duckweed MiAMP1 proteins warrant further investigation against some of the known fungal and oomycetes pathogens of duckweed (Fisch, 1884; Gaumann, 1928; Vanky, 1981; Rejmankova *et al*., 1986; Flaishman *et al*., 1997; Czeczuga *et al*., 2005; Brand *et al*., 2021). Other commonly differentially expressed protein families were associated with cytochrome P450s and phytohormones, indicating that specialized metabolites may have an important role in duckweeds infection response. Together, our data highlights that, despite the absence of *EDS1*, there are conserved areas of immune response pathways across plant species, such as phytohormones and secondary metabolism. Perhaps the reduced reliance on NLR–EDS1 pathways in duckweeds released selective constraints allowing avoidance of the typical plant–NLR vs pathogen–effector arms races and facilitating amplification of alternate immune strategies.

Duckweeds are an exciting system for research into plant immunity. They provide a reduced redundancy system in terms of both gene copy number and pathways present. In addition, their rapid lifecycle of just 34 h (Michael *et al*., 2020), small size and susceptibility to model pathogens make them the perfect system for high‐throughput experimentation. The genomic and genetic resources are also developing at a rapid pace (Wang *et al*., 2014; Michael *et al*., 2017, 2020; Hoang *et al*., 2018; An *et al*., 2019; Ho *et al*., 2019; Xu *et al*., 2019; Harkess *et al*., 2021) and a range of transformation protocols (Ko *et al*., 2011; J. Yang *et al*., 2018; G‐L. Yang *et al*., 2018; Liu *et al*., 2019; Acosta *et al*., 2021; Chanroj *et al*., 2021). Many interesting questions remain to be answered including how duckweed plants can thrive in a wide range of environments despite being highly susceptible to bacterial phytopathogens in the laboratory setting. One hypothesis is that duckweeds may be using means of disease protection that are inherently absent in the laboratory due to the way duckweeds are propagated. This may include microbiome‐mediated disease protection, small peptide defense molecules such as MiAMP1s or chemical defense strategies. A combination of genetics, molecular biology and metabolomic approaches will be needed to address these hypotheses.

## Author contributions

ELB performed genomic/transcriptomic presence absence analysis, growth curves, hormone assays, microscopy and RNA‐Seq analysis. MBT and ELB performed the screen of phytopathogens across duckweed species and RNA extractions. BWA and TPM performed DNA extraction, genome assembly and annotation of *L. punctata* 5635 genome. ELB and KVK designed the study. ELB prepared figures and wrote the full manuscript draft; all authors have read and approved the final version of the manuscript.

## Supporting information


**Dataset S1** Powerpoint presentation of pathogen inoculation symptoms.
**Dataset S2** Z‐stack microscopy.
**Dataset S3** Differentially expressed gene tables *Spirodela polyrhiza*.
**Dataset S4** Differentially expressed gene tables *Landoltia punctata*.
**Dataset S5** Orthogroup assignment and overlap across pathogen treatments and species.

**Fig. S1** PlotMDS for all *Spirodela polyrhiza* RNA‐Seq samples before outlier removal.
**Fig. S2** PlotMDS for *Spirodela polyrhiza* RNA‐Seq samples after outlier removal.
**Fig. S3** PlotMDS for all *Landoltia punctata* RNA‐Seq samples before outlier removal.
**Fig. S4** PlotMDS for *Landoltia punctata* RNA‐Seq samples after outlier removal.
**Fig. S5** Phylogeny of Nucleotide‐binding Leucine‐rich repeat Receptor proteins in duckweeds.
**Fig. S6** Phylogeny of receptor‐like kinase proteins in duckweeds.
**Fig. S7** Phylogeny of receptor‐like protein type proteins in duckweeds.
**Fig. S8** Phylogeny of MiAMP1 domain‐containing proteins in duckweed species.
**Fig. S9** Symptoms of *Spirodela polyrhiza* populations derived from a single mother frond after infection with *Pst* DC3000.
**Fig. S10** Microscopy of frond surface 5 d post flood inoculation with *Pseudomonas syringae* pv *tomato* DC3000.
**Fig. S11** Microscopy of duckweed frond surface 5 and 7 d post flood inoculation with *Pseudomonas syringae* pv *tomato* DC3000 hrcC.
**Fig. S12** Low bacterial load infection of *Landoltia punctata* 1 month post inoculation.
**Fig. S13** Role of coronatine in *Pst* DC3000 infection of *Spirodela polyrhiza*.
**Fig. S14** Role of coronatine in *Pst* DC3000 infection of *Spirodela polyrhiza*.
**Fig. S15** Salicylic acid phytotoxicity to *Spirodela polyrhiza* upon buffer or *Pst* DC3000 treatment.
**Fig. S16** Dissecting microscope images of duckweed 12 d post inoculation with salicylic acid.
**Fig. S17** Role of salicylic acid in *Pst* DC3000 infection of *Spirodela polyrhiza*.
**Fig. S18** Role of salicylic acid in *Pst* DC3000 infection of *Spirodela polyrhiza*.
**Fig. S19** Role of salicylic acid in *Pst* DC3000 infection of *Spirodela polyrhiza*.
**Fig. S20** Role of salicylic acid in *Pst* DC3000 infection of *Spirodela polyrhiza*.
**Fig. S21** Role of salicylic acid in *Pss* B728a infection of *Landoltia punctata*.
**Fig. S22** Role of salicylic acid in *Pss* B728a infection of *Landoltia punctata*.
**Fig. S23** Bar chart of number of genes differentially expressed upon bacterial treatments.
**Fig. S24** Log_2_ fold change of selected gene families following bacterial pathogen exposure of duckweeds.
**Fig. S25** Log_2_ fold change of homologs of *Arabidopsis thaliana* bacterial responsive genes.
**Fig. S26** Differential expression upon pathogen treatment of the duckweed homologs of Arabidopsis salicylic acid marker genes.
**Fig. S27** Orthogroups with conserved upregulation upon pathogen treatment of duckweed species and the expression of orthologs in *Arabidopsis thaliana*.
**Fig. S28** Heatmap displaying log_2_ fold change of rice homologs of duckweed conserved upregulated gene upon pathogen infection.

**Table S1** Genome assembly statistics for *Landoltia punctata* 5635 genome and proteome Busco scores.
**Table S2** Table of common name and duckweed gene IDs homologous to *Arabidopsis thaliana* disease marker genes.
**Table S3** Copy number of orthologs/homologs of immune associated genes.
**Table S4** Table of *Spirodela polyrhiza* homologs to Arabidopsis immunity genes.
**Table S5** Syndrome prevalence upon pathogen treatment of Lemnaceae species.
**Table S6** Log_2_ fold change of JAZ domain‐containing *Spirodela polyrhiza* genes.
**Table S7** Log_2_ fold change of WRKY domain‐containing *Spirodela polyrhiza* genes.
**Table S8** Log_2_ fold change of MiAMP1 domain‐containing *Spirodela polyrhiza* genes.
**Table S9** Log_2_ fold change of NB‐ARC domain‐containing *Spirodela polyrhiza* genes.
**Table S10** Log_2_ fold change of JAZ domain‐containing *Landoltia punctata* genes.
**Table S11** Log_2_ fold change of WRKY domain‐containing *Landoltia punctata* genes.
**Table S12** Log_2_ fold change NBARC domain‐containing genes *Landoltia punctata*.
**Table S13** Log_2_ fold change MiAMP1 domain‐containing genes *Landoltia punctata*.
**Table S14** Orthologs to *Arabidopsis thaliana* marker genes that are significantly upregulated upon *Pst* treatment log_2_FC > 0.75 FDR < 0.05 in either *Spirodela polyrhiza* or *Landoltia punctata*.
**Table S15** Homologs to *Arabidopsis thaliana* marker genes that are significantly upregulated upon *Pst* treatment log_2_FC > 1 FDR < 0.05.
**Table S16** Table of duckweed genes homologous of *Arabidopsis thaliana* salicylic acid responsive genes.
**Table S17** Log_2_ fold change of *Spirodela polyrhiza* genes homologous to *Arabidopsis thaliana* salicylic acid responsive genes.
**Table S18** Log_2_ fold change of *Landoltia punctata* genes homologs to *Arabidopsis thaliana* salicylic acid responsive genes.
**Table S19** Orthogroups upregulated in *Landoltia punctata* and *Spirodela polyrhiza* after *Pst* DC3000 treatment.
**Table S20** Log_2_ fold change of rice genes homologous to pathogen upregulated duckweed genes.
Please note: Wiley Blackwell are not responsible for the content or functionality of any Supporting Information supplied by the authors. Any queries (other than missing material) should be directed to the *New Phytologist* Central Office.

## Data Availability

Sequencing reads for *S. polyrhiza* and *L. punctata* RNA‐Seq can be found at the respective NCBI BioProjects PRJNA808038 and PRJNA801691. The *L. punctata* 5635 genome can be found on CoGe accession ID: 63585. Scripts for analysis presented in the manuscript can be found on the github repository https://github.com/erin‐baggs/DuckweedRNA. Z‐stack of *Pst* DC3000 infection of *Spirodela polyrhiza* is available from Zenodo doi: 10.5281/zenodo.5639580.
