# Supplementary material for: Characterization of defense responses against bacterial pathogens in duckweeds lacking EDS1
Source: New Phytol. 2022 Sep 25;236(5):1838–55. doi: 10.1111/nph.18453 (PMC9828482; doi:10.1111/nph.18453)
Supplement: Supplementary file 1 — Dataset S1 Powerpoint presentation of pathogen inoculation symptoms. Dataset S2 Z‐stack microscopy. Dataset S3 Differentially expressed gene tables Spirodela polyrhiza. Dataset S4 Differentially expressed gene tables Landoltia punctata. Dataset S5 Orthogroup assignment and overlap across pathogen treatments and species. [file NPH-236-1838-s002.pdf]

New Phytologist **Supporting Information**

Article title: **Characterization of defense responses against bacterial pathogens in duckweeds lacking EDS1.**

Authors: *Baggs E.L, Tiersma M.B, Abramson B.W , Michael T.P and Krasileva K.V*

Article acceptance date: 19 August 2022

The following supplemental datasets is available for this article:

Supplemental datasets

Supplemental datasets are available at github (https://github.com/krasileva-group/Thesis-Scripts) or zenodo (links below).

Dataset S1 - Powerpoint presentation of pathogen inoculation symptoms

Available on Zenodo at doi : 10.5281/zenodo.5733456.

A compilation of images displaying the range of symptoms observed when *Spirodela polyrhiza, Landoltia punctata* and *Wolffia australiana* are treated with *Pseudomonas* and *Xanthomonas* species phytopathogens.

Dataset S2 – Z-stack microscopy

Available at A: https://doi.org/10.5281/zenodo.5639580 and B,C: https://doi.org/10.5281/zenodo.6803451.

A. Confocal microscopy Z-stack time course of *S. polyrhiza* frond 10 days post flood inoculated with *Pst* DC3000 stained with SytoBC. False colors: pink - *Pst* DC3000, green - plastids, gray - transmitted light. Scale bar is in top right corner at 20 μm. B. Confocal microscopy Z-stack time course of *S. polyrhiza* frond 5 days post flood inoculation with *Pst* DC3000 hrcC-. Images start at frond surface. Images were taken with a 63x objective, green coloring represents *Pst* DC3000 hrcC- stained with SytoBC, in addition the guard cell wall appears as a green ellipse due to autofluorescence, red indicates chlorophyll fluorescence. Scale bar is 5 μm. C. Setup as in B. except at 7 days post inoculation with *Pst* DC3000 hrcC-.

Dataset S3 - Differentially expressed gene tables *S. polyrhiza*

Available at https://github.com/krasileva-group/Thesis-Scripts/blob/7c37e65a1a4328ba95b72b11b99a780158e961f0/Sdata3.xlsx

Shorthand labeling scheme used throughout is outlined in Table 0 – contents.

Table 1 – Raw numbers for the waterfall plot.

Tables 2-11 – Tables for each comparison between treatments containing genes which passed the cutoff criteria for differential expression along. Table includes values for log_2FC, logCPM, LR, Pvalue and FDR from edgeR.

Tables 12-31 – Tables of significantly up or down regulated genes between each treatment comparison alongside metadata about gene common names and protein domains.

Table 32 – Table of all annotations associated with upregulated genes across all timepoints. Color coded by time of differential expression, grey – 30 min, green – 1hr, yellow – 6 hrs , orange 12 hrs.

Tables 33-37 Tables containing *Spirodela polyrhiza* gene identifiers for WRKY, NLR, JAZ and MiAMP1 proteins.

Table 38 – Query terms used to identify proteins after Pfamscan which belong to WRKY, NLR, JAZ and MiAMP1 protein families.

Dataset S4 - Differentially expressed gene tables *L. punctata*

Available at https://github.com/krasileva-group/Thesis-Scripts/blob/7c37e65a1a4328ba95b72b11b99a780158e961f0/Sdata4-DE-Lpunctata.xlsx

Shorthand labeling scheme used throughout is outlined in Table 0 – contents.

Tables 1-8 – Tables for each comparison between treatments containing genes which passed the cutoff criteria for differential expression along. Table includes values for log_2FC, logCPM, LR, Pvalue and FDR from edgeR.

Dataset S5 - Orthogroup assignment and overlap across pathogen treatments and species

Available at https://github.com/krasileva-group/Thesis-Scripts/blob/7c37e65a1a4328ba95b72b11b99a780158e961f0/Sdata5-orthogroup-across-

treatment-analysus.xlsx

Table 1	Total numbers of orthogroups falling within possible combinations of upregulation across treatments $log_2FC > 0.05$ FDR < 0.05.
Table 2	Orthogroups upregulated in both *Spirodela Pst* DC3000 and *Landoltia Pst* DC3000 treatment
Table 3	Orthogroups upregulated in *Spirodela polyrhiza* buffer vs *Pst* DC3000 / *Pst* DC3000 hrcC and *Landoltia Pss* B728a treatments
Table 4	Orthogroups upregulated in *Spirodela polyrhiza* buffer vs *Pst* DC3000 and *Landoltia Pst* DC3000 and not upregulated in *Spirodela polyrhiza Pst* DC3000 hrcC
Table 5	Orthogroups upregulated in *Spirodela polyrhiza* buffer vs *Pst* DC3000 / *Pst* DC3000 hrcC and *Landoltia Pst* DC3000 and *Landoltia Pss* B728a
Table 6	Orthogroups upregulated *in Spirodela polyrhiza* and *Landoltia* buffer vs *Pst* DC3000 but not *S. polyrhiza Pst* DC3000 hrcC
Table 7	Orthogroups upregulated in either *Landoltia* buffer vs *Pst* DC3000 *or Landoltia Pss* B728a
Table 8	Orthogroups upregulated in *Spirodela polyrhiza* buffer vs *Pst* DC3000 / *Pst* DC3000 hrcC
Table 9	Orthogroups upregulated in *Landoltia Pss* B728a and *Landoltia Pst* DC3000
Table 10	Orthogroups upregulated in *Spirodela polyrhiza Pst* DC3000 and *Landoltia Pss* B728a
Table 11	Orthogroups upregulated in *Spirodela polyrhiza Pst* DC3000 hrcC and *Landoltia Pst* DC3000 and *Landoltia Pss* B728a
Table 12	Orthogroups upregulated in *Spirodela polyrhiza Pst* DC3000 and *Spirodela polyrhiza Pst* DC3000 hrcC and *Landoltia Pss* B728a
Table 13	Orthogroups upregulated in *Spirodela polyrhiza Pst* DC3000 and *Spirodela polyrhiza Pst* DC3000 hrcC and *Landoltia Pst* DC3000
Table 14	Orthogroups upregulated in *Spirodela polyrhiza Pst* DC3000 and *Landoltia Pst* DC3000
Table 15	Orthogroups upregulated in *Spirodela polyrhiza Pst* DC3000 hrcC and *Landoltia Pss* B728a
Table 16	Orthogroups upregulated in *Spirodela polyrhiza Pst* DC3000 hrcC
Table 17	Orthogroups upregulated in *Spirodela polyrhiza Pst* DC3000
Table 18	Orthogroups upregulated in *Landoltia Pst* DC3000
Table 19	Orthogroups upregulated in *Landoltia Pss* B728a
Table 20	Orthogroups upregulated in *Spirodela polyrhiza Pst* DC3000 hrcC and *Landoltia Pst* DC3000
