# Supplementary material for: Characterization of defense responses against bacterial pathogens in duckweeds lacking EDS1
Source: New Phytol. 2022 Sep 25;236(5):1838–55. doi: 10.1111/nph.18453 (PMC9828482; doi:10.1111/nph.18453)
Supplement: Supplementary file 2 — Fig. S1 PlotMDS for all Spirodela polyrhiza RNA‐Seq samples before outlier removal. Fig. S2 PlotMDS for Spirodela polyrhiza RNA‐Seq samples after outlier removal. Fig. S3 PlotMDS for all Landoltia punctata RNA‐Seq samples before outlier removal. Fig. S4 PlotMDS for Landoltia punctata RNA‐Seq samples after outlier removal. Fig. S5 Phylogeny of Nucleotide‐binding Leucine‐rich repeat Receptor proteins in duckweeds. Fig. S6 Phylogeny of receptor‐like kinase proteins in duckweeds. Fig. S7 Phylogeny of receptor‐like protein type proteins in duckweeds. Fig. S8 Phylogeny of MiAMP1 domain‐containing proteins in duckweed species. Fig. S9 Symptoms of Spirodela polyrhiza populations derived from a single mother frond after infection with Pst DC3000. Fig. S10 Microscopy of frond surface 5 d post flood inoculation with Pseudomonas syringae pv tomato DC3000. Fig. S11 Microscopy of duckweed frond surface 5 and 7 d post flood inoculation with Pseudomonas syringae pv tomato DC3000 hrcC. Fig. S12 Low bacterial load infection of Landoltia punctata 1 month post inoculation. Fig. S13 Role of coronatine in Pst DC3000 infection of Spirodela polyrhiza. Fig. S14 Role of coronatine in Pst DC3000 infection of Spirodela polyrhiza. Fig. S15 Salicylic acid phytotoxicity to Spirodela polyrhiza upon buffer or Pst DC3000 treatment. Fig. S16 Dissecting microscope images of duckweed 12 d post inoculation with salicylic acid. Fig. S17 Role of salicylic acid in Pst DC3000 infection of Spirodela polyrhiza. Fig. S18 Role of salicylic acid in Pst DC3000 infection of Spirodela polyrhiza. Fig. S19 Role of salicylic acid in Pst DC3000 infection of Spirodela polyrhiza. Fig. S20 Role of salicylic acid in Pst DC3000 infection of Spirodela polyrhiza. Fig. S21 Role of salicylic acid in Pss B728a infection of Landoltia punctata. Fig. S22 Role of salicylic acid in Pss B728a infection of Landoltia punctata. Fig. S23 Bar chart of number of genes differentially expressed upon bacterial treatments. Fig. S24 Log2 fold ch [file NPH-236-1838-s003.pdf]

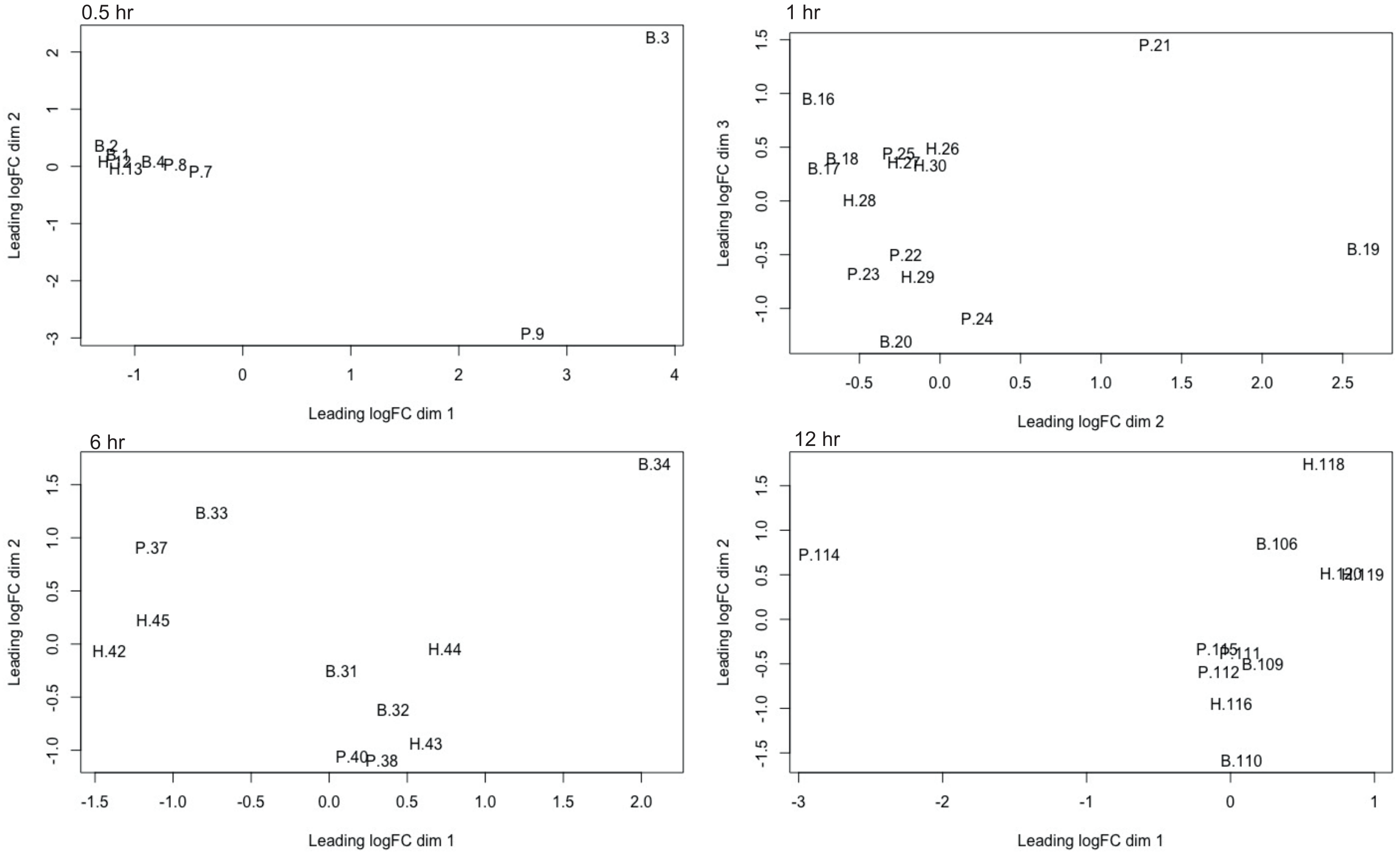

Fig. S1. PlotMDS for all *S. polyrhiza* RNAseq samples before outlier removal.
The PlotMDS dimensions that are shown are those that best separated the treatments. Initial represents treatment condition, B = Buffer, P = *Pst* DC3000 and H= *Pst* DC3000 hrcC.

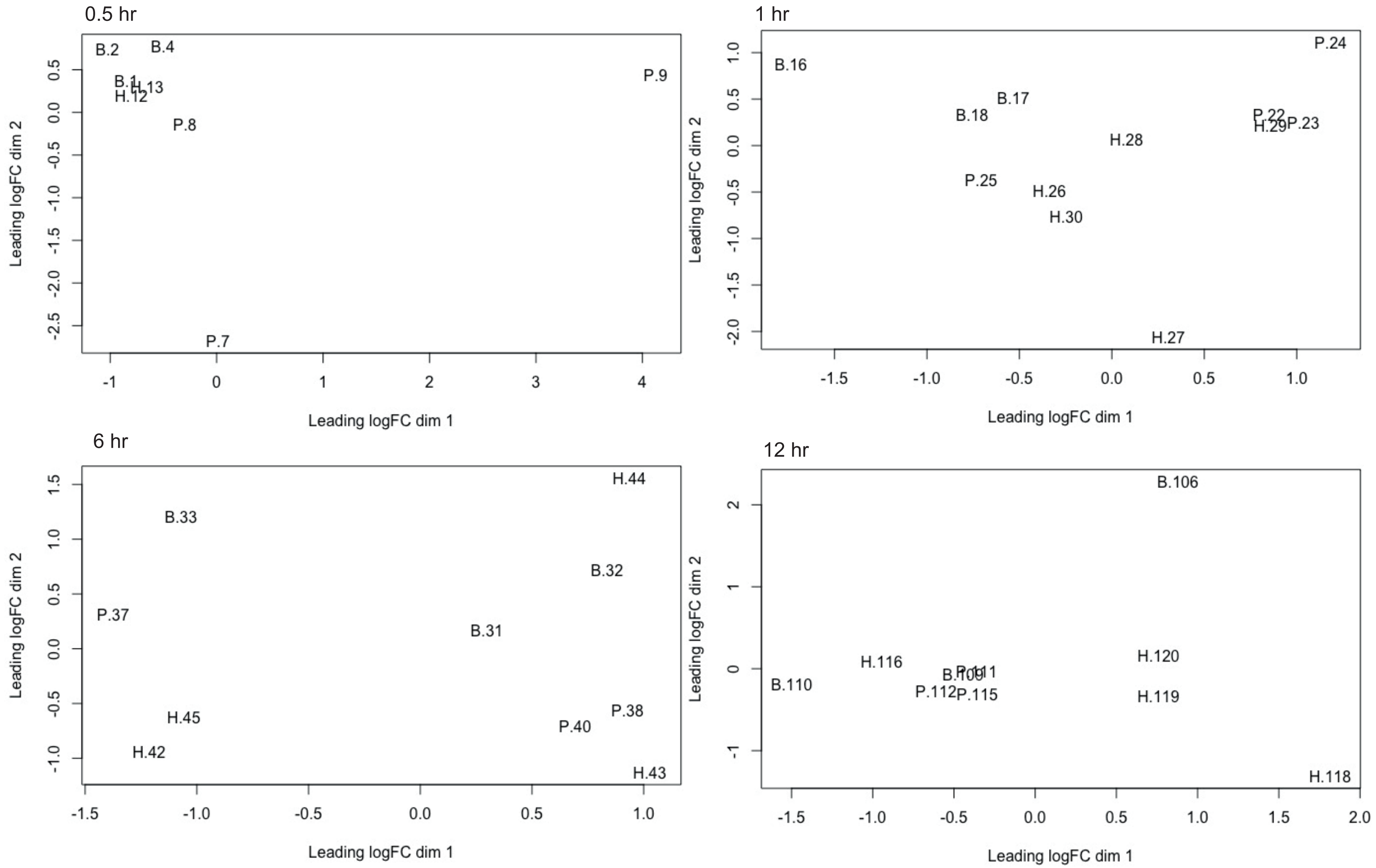

Fig. S2. PlotMDS for *S. polyrhiza* RNAseq samples after outlier removal.
PlotMDS for *S. polyrhiza* RNAseq samples after outlier removal dimensions shown are those that best separates the treatments. Initial represents treatment condition, B = Buffer, P = *Pst* DC3000 and H= *Pst* DC3000 hrcC.

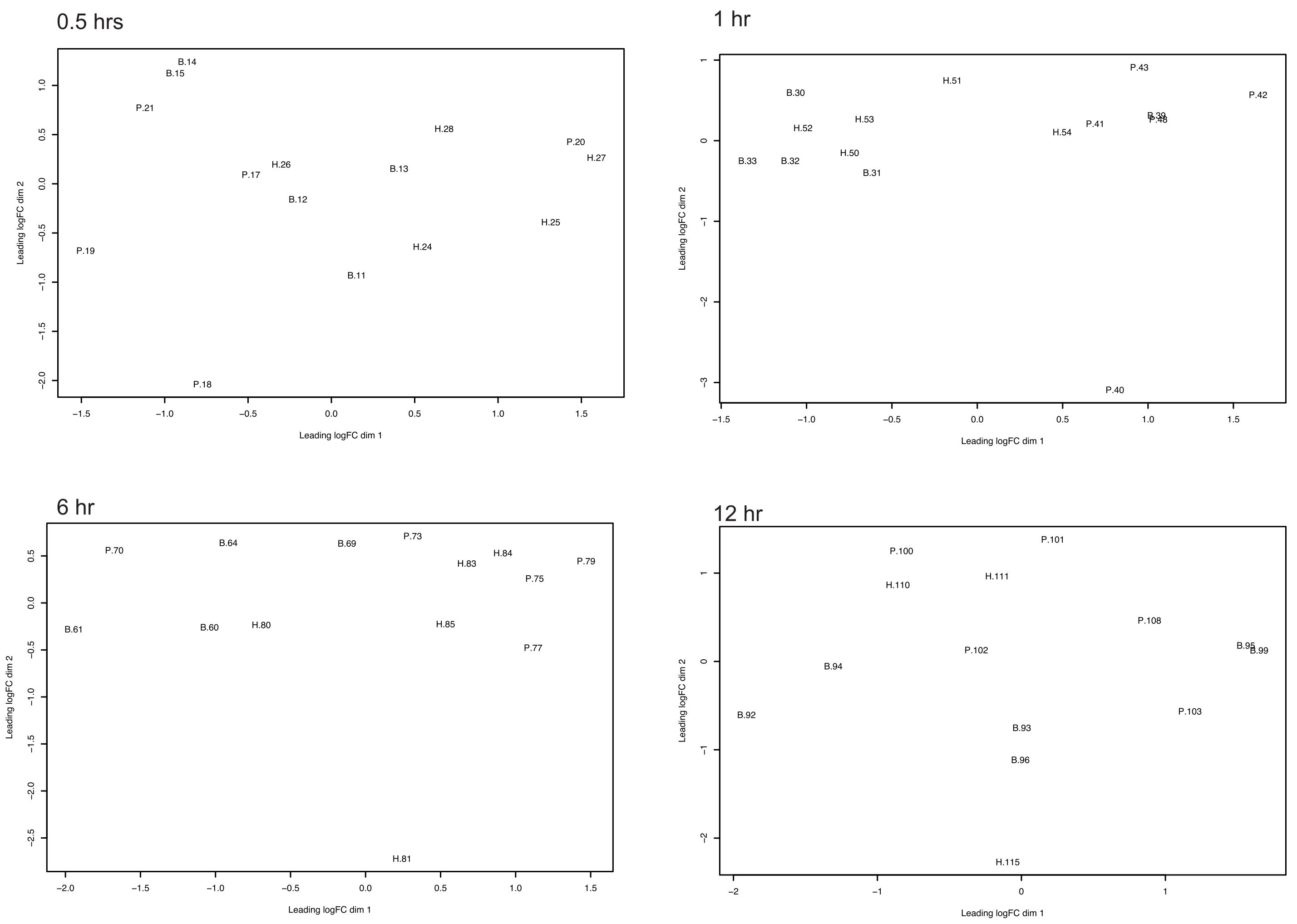

Fig. S3. PlotMDS for all *L. punctata* RNAseq samples before outlier removal.
PlotMDS dim=c(1,2) of all *L. punctata* RNAseq samples for all samples prior to outlier removal. The B prefix indicates buffer H indicates *Pss* B728a and P indicates *Pst* DC3000.

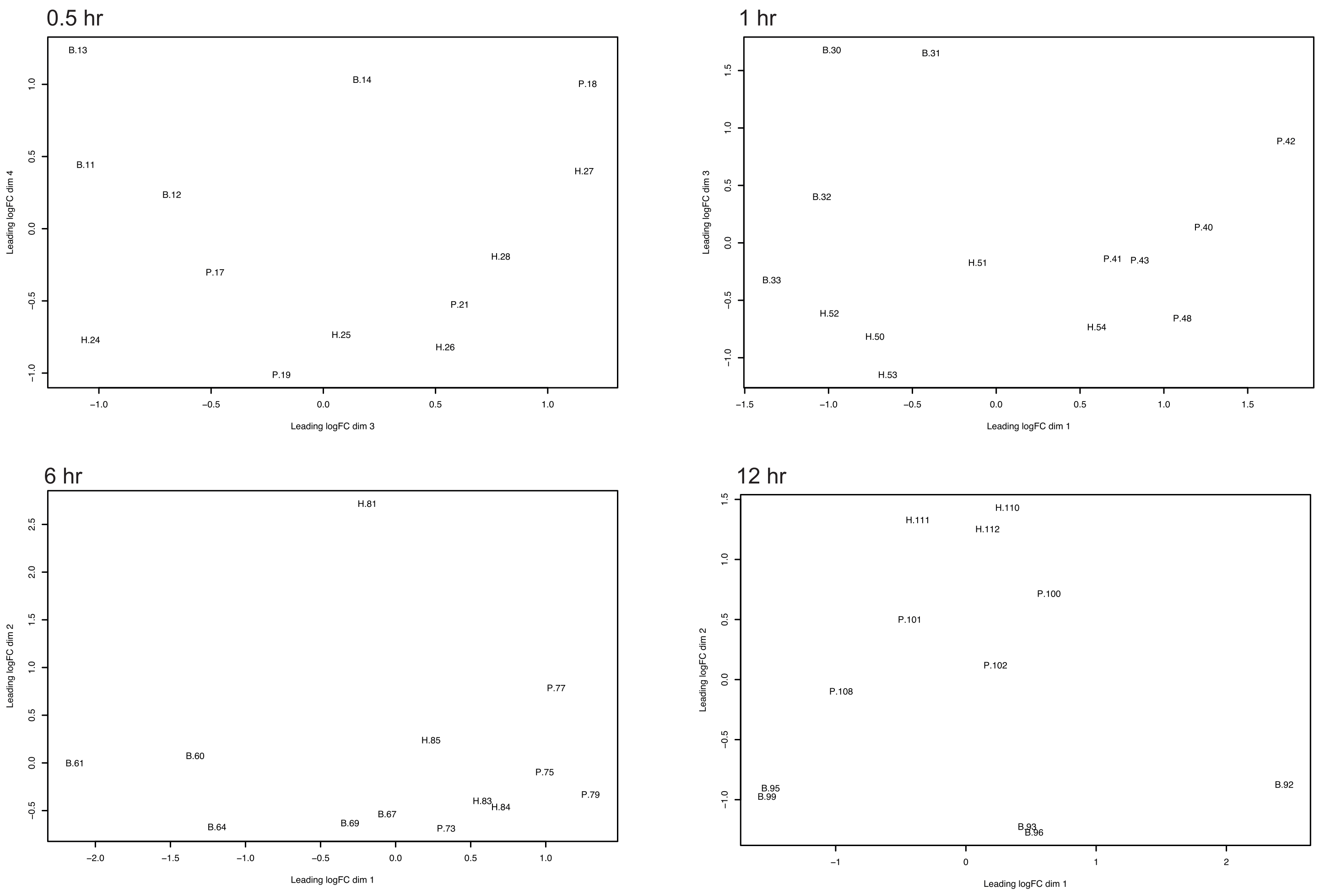

Fig. S4. PlotMDS for *L. punctata* RNAseq samples after outlier removal.
PlotMDS of *L. punctata* RNAseq samples after outlier removal. Dimensions displayed are those that best separate treatments. The B prefix indicates buffer, H indicates *Pss* B728a, and P indicates *Pst* DC3000.

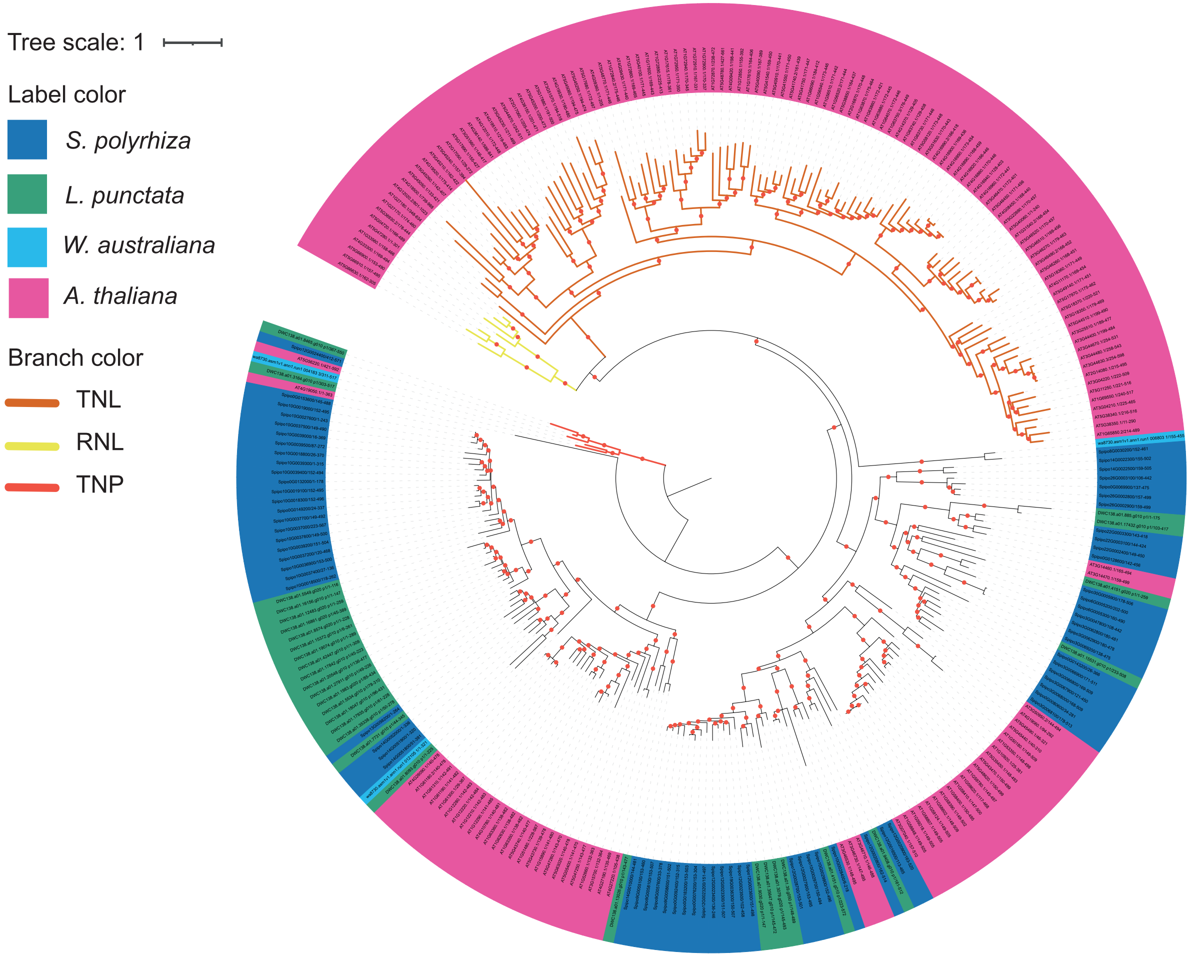

Fig. S5. Phylogeny of NLR proteins in duckweeds.
Maximum likelihood phylogeny of NB-ARC proteins (Pfam PF00931) from *A. thaliana, S. polyrhiza, L. punctata* and *W. australiana*. Alignments were manually curated for the presence of NB-ARC functional motifs. Red dots indicate bootstrap values >70. Orange branches indicate TIR-NBARC-LRR proteins, red branches indicate TIR-NBARC-like-TPR repeat containing proteins and yellow branches indicate RPW8-NB-ARC-LRR (RNLS). Gene identifiers are colored to indicate species: *S. polyrhiza* - dark blue, *W. australiana* - light blue, *L. punctata* - green and *A. thaliana* - pink. Tree rooted on TNPs.

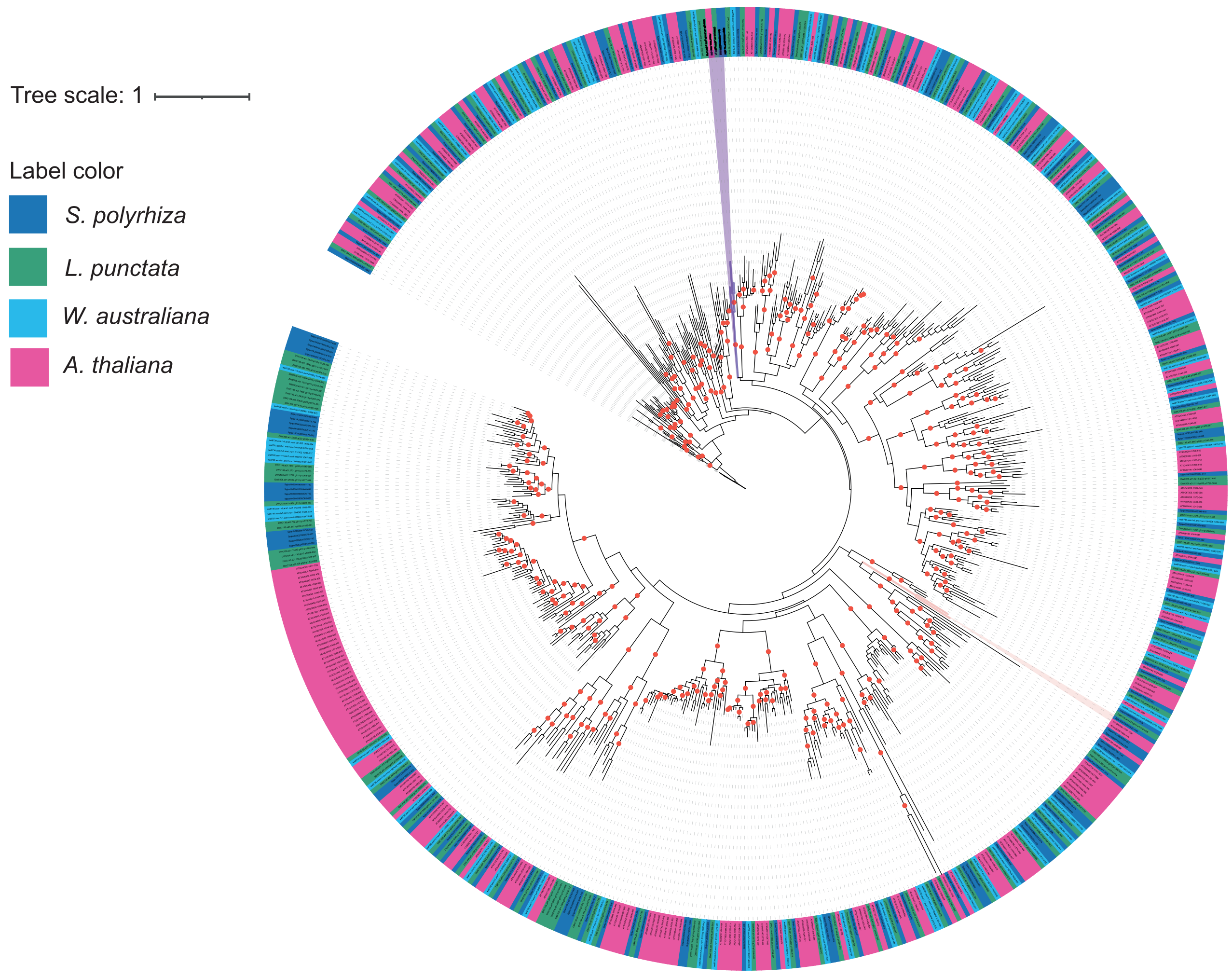

Fig. S6. Phylogeny of RLK proteins in duckweeds.
Maximum likelihood phylogeny of RLK proteins from *A. thaliana, S. polyrhiza, L. punctata* and *W. australiana*. Alignments were manually curated for the presence of RLK functional motifs. Red dots indicate bootstrap values >70. Tree rooted at midpoint. Gene identifiers are colored to indicate species: *S. polyrhiza* - dark blue, *W. australiana* - light blue, *L. punctata* - green and *A. thaliana* - pink. Purple range indicates SOBIR1 clade and pink range indicates FLS2 clade.

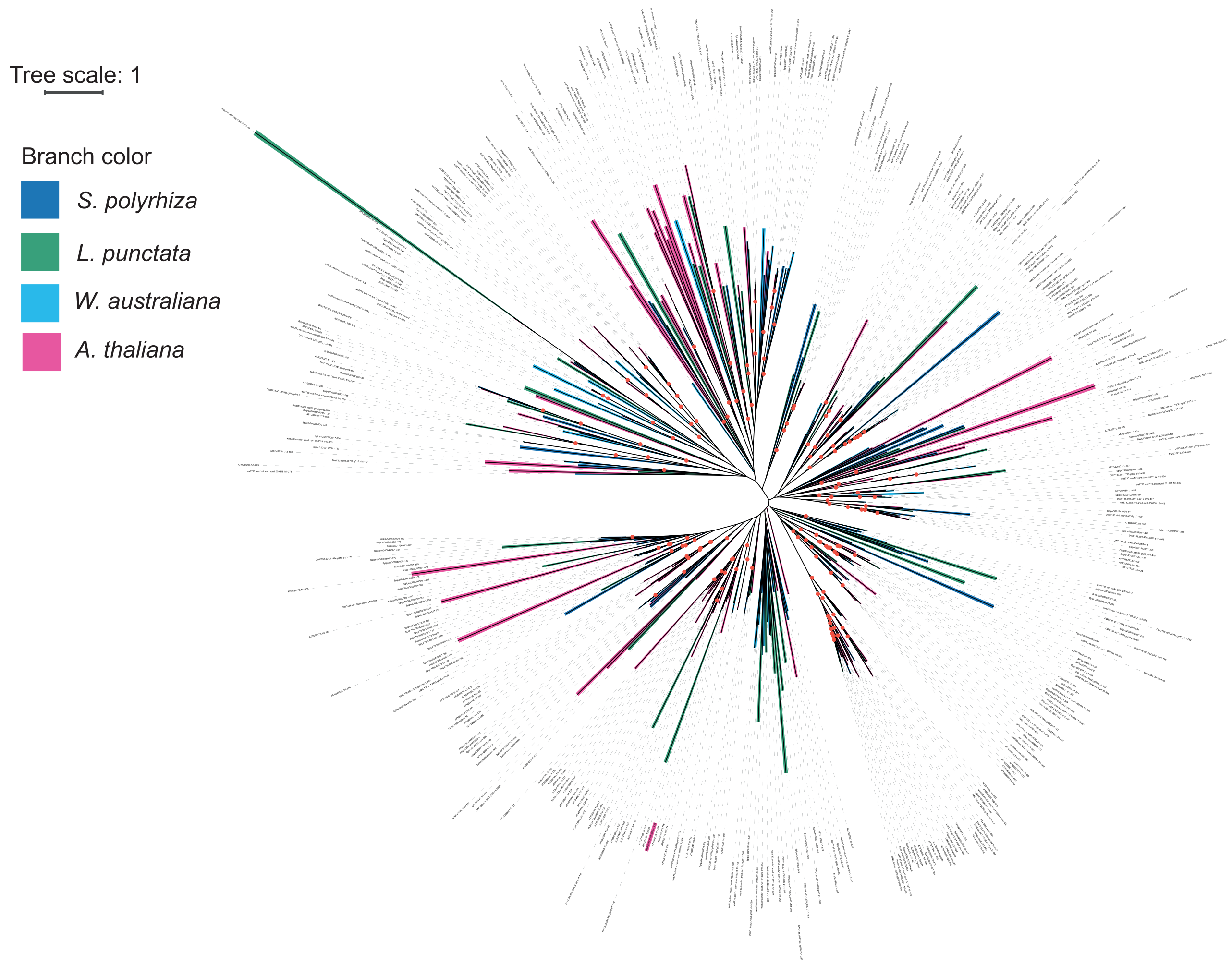

Fig. S7. Phylogeny of RLP type proteins in duckweeds.
Maximum likelihood phylogeny of RLP proteins from *A. thaliana*, *S. polyrhiza*, *L. punctata* and *W. australiana*. Alignments were manually curated for conserved motifs. Red dots indicate bootstrap values >70. Branches are colored to indicate species: *S. polyrhiza* - dark blue, *W. australiana* - light blue, *L. punctata* - green and *A. thaliana* - pink.

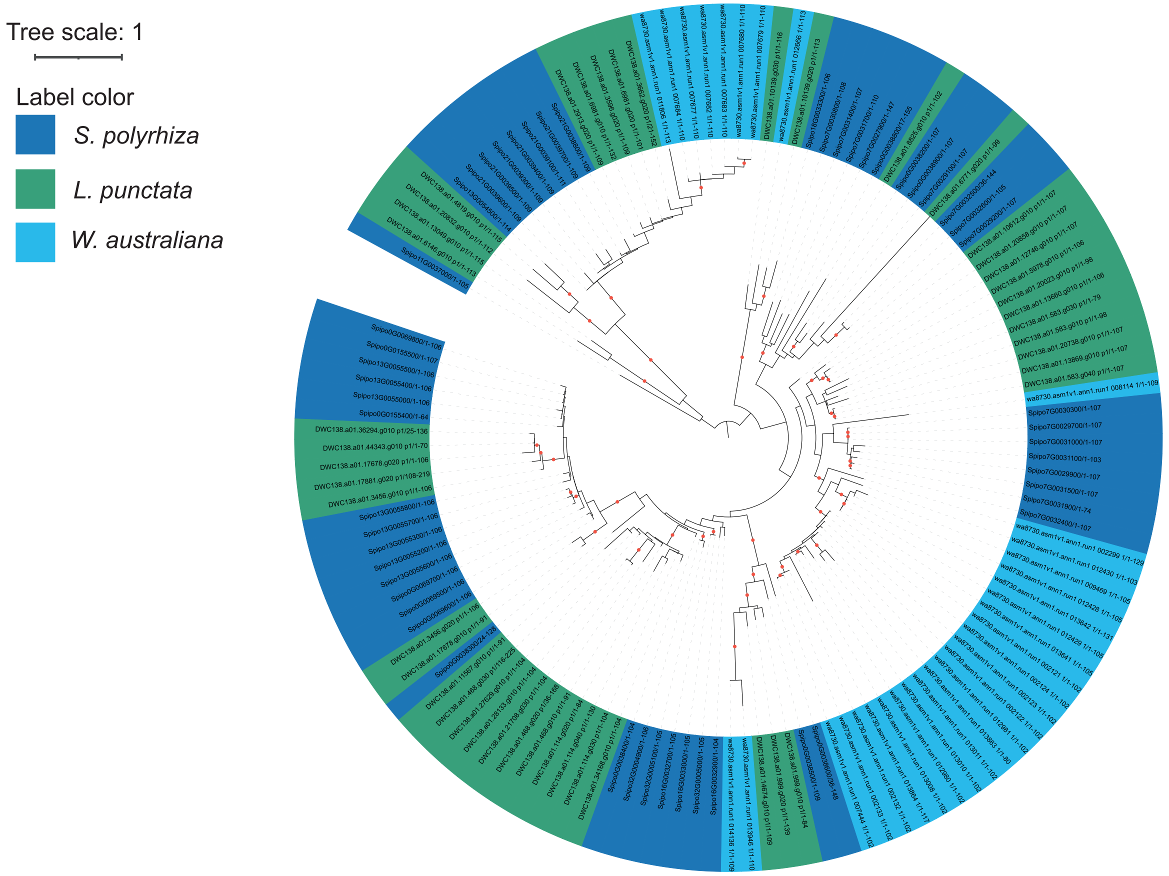

Fig. S8. Phylogeny of MiAMP1 domain containing proteins in duckweed species.
Maximum likelihood phylogeny of MiAMP1 (Pfam PF09117) domain containing proteins from *S. polyrhiza*, *L. punctata* and *W. australiana*. Red dots indicate bootstrap values >70. Tree rooted at midpoint. Gene identifiers are colored to indicate species: *S. polyrhiza* - dark blue, - *W. australiana* - light blue and *L. punctata* - green.

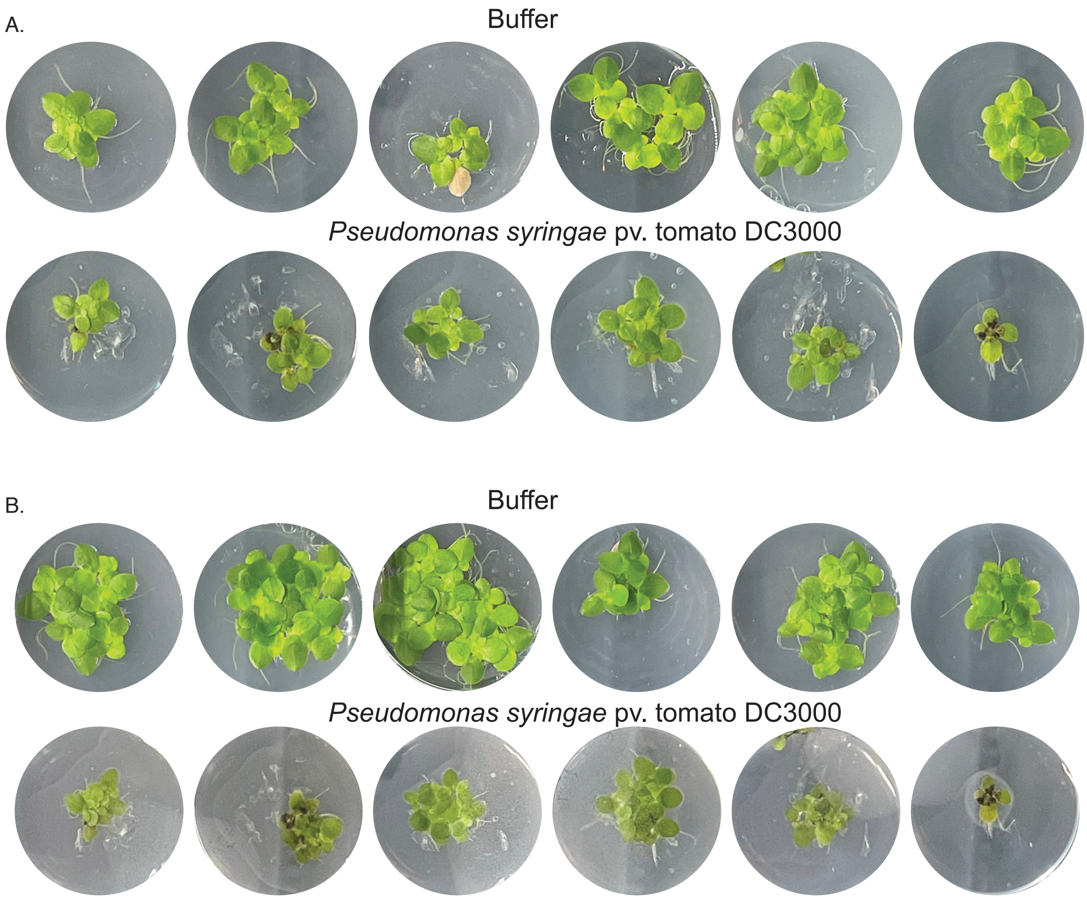

Figure S9. Symptoms of *S. polyrhiza* populations derived from a single mother frond after infection with *Pst* DC3000 .
A. Images of *S. polyrhiza* fronds 10pi of a single frond with *Pst* DC3000. B. 15 dpi other-wise as in A.

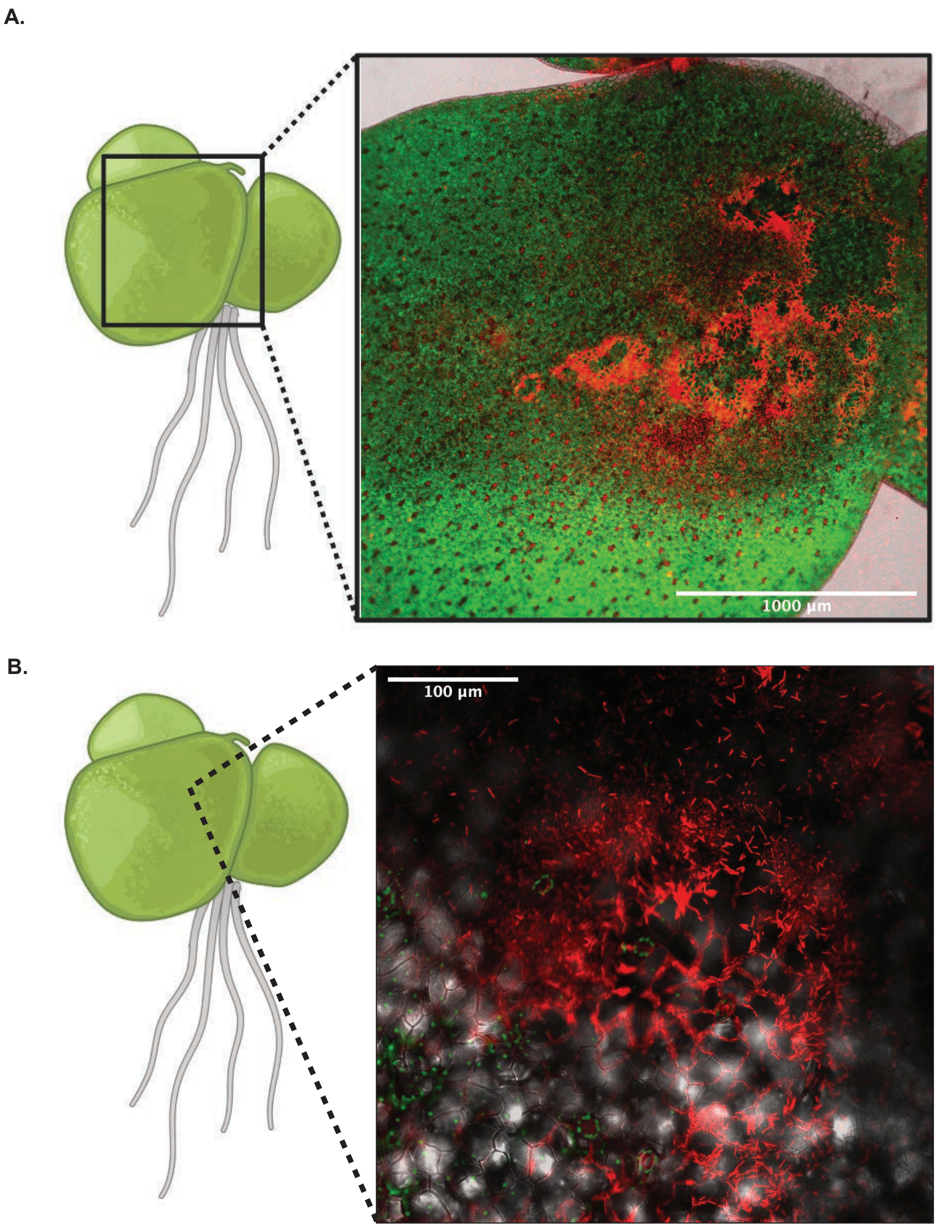

Fig. S10 Microscopy of frond surface 5 days post flood innoculation with *Pseudomonas syringae* pv. tomato DC3000.
A. Confocal microscopy 5x magnification red false coloring represents *Pst* DC3000 stained with SytoBC, green shows chlorophyll fluorescence and grey is transmitted light. B. 20x confocal microscopy of concentrated *Pst* DC3000 populations.

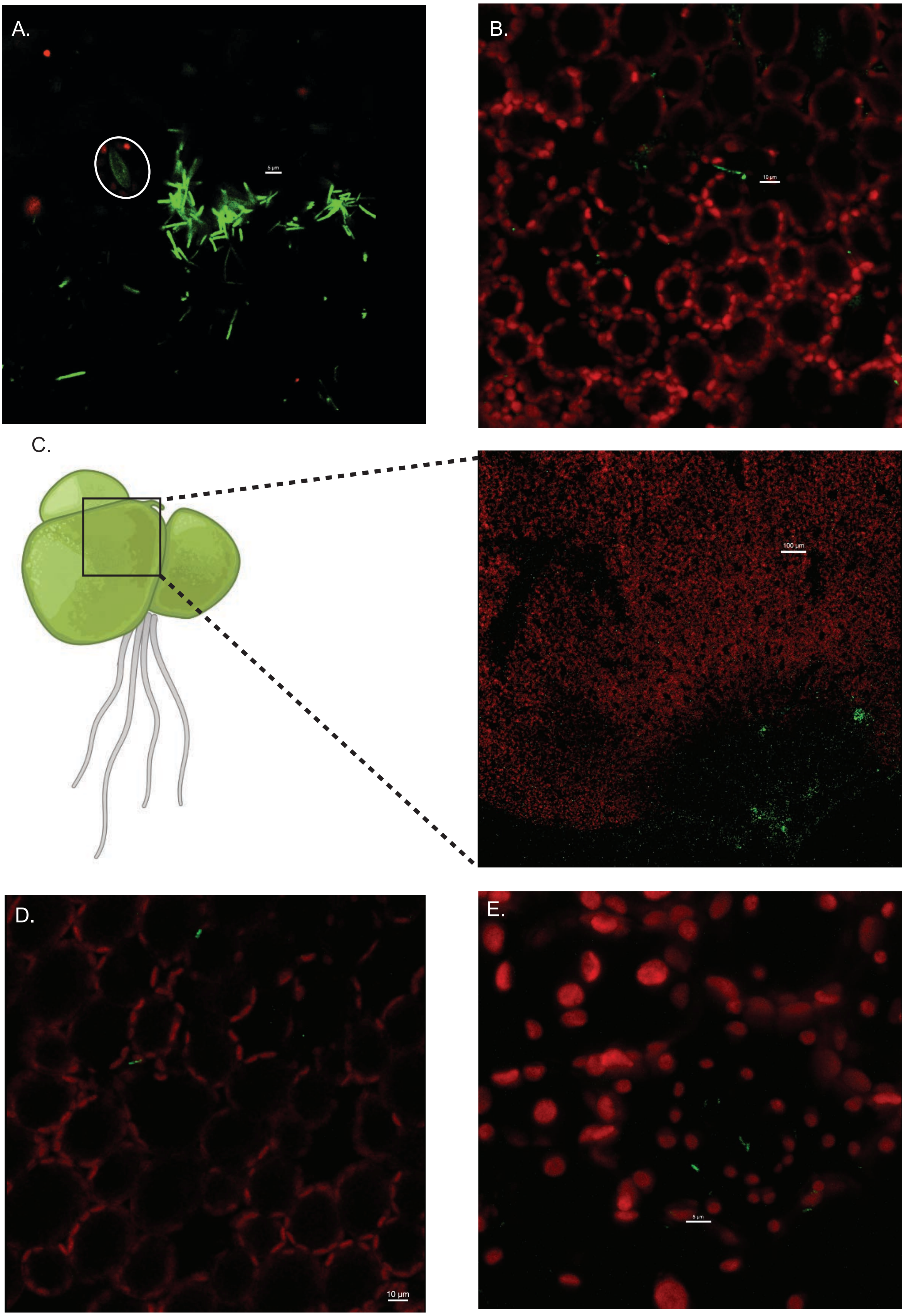

Fig. S11. Microscopy of duckweed frond surface five and seven days post flood inoculation with *Pseudomo-nas syringae* pv. tomato DC3000 *hrcC*.
A. Confocal microscopy of *S. polyrhiza* frond 5 dpi with *Pst* DC3000 *hrcC*. Image is of frond surface at 63x objective, green coloring represents *Pst* DC3000 *hrcC* stained with SytoBC, red shows chlorophyll fluorescence. White circle highlights the autofluorescent of guard cell walls. Scale bar is 5 µm B. Image of *Pst* DC3000 *hrcC* within the mesophyll at 5 dpi, with coloring as above 20x objective. Scale bar is 10 µm. C. Confocal microscopy of *S. polyrhiza* frond 7 dpi with *Pst* DC3000 *hrcC*. Image is at 5x magnification, green coloring represents *Pst* DC3000 *hrcC* stained with SytoBC, red shows chlorophyll fluorescence. Scale bar is 100 µm D. Confocal microscopy of *S. polyrhiza* frond 7 dpi with *Pst* DC3000 *hrcC*. Image is of frond surface at 20x magnification, green coloring represents *Pst* DC3000 *hrcC* stained with SytoBC, red shows chlorophyll fluorescence. Scale bar is 10 µm. E. Image of *Pst* DC3000 *hrcC* within the mesophyll at 7dpi, with coloring as above and100x objective. Scale bar is 5 µm.

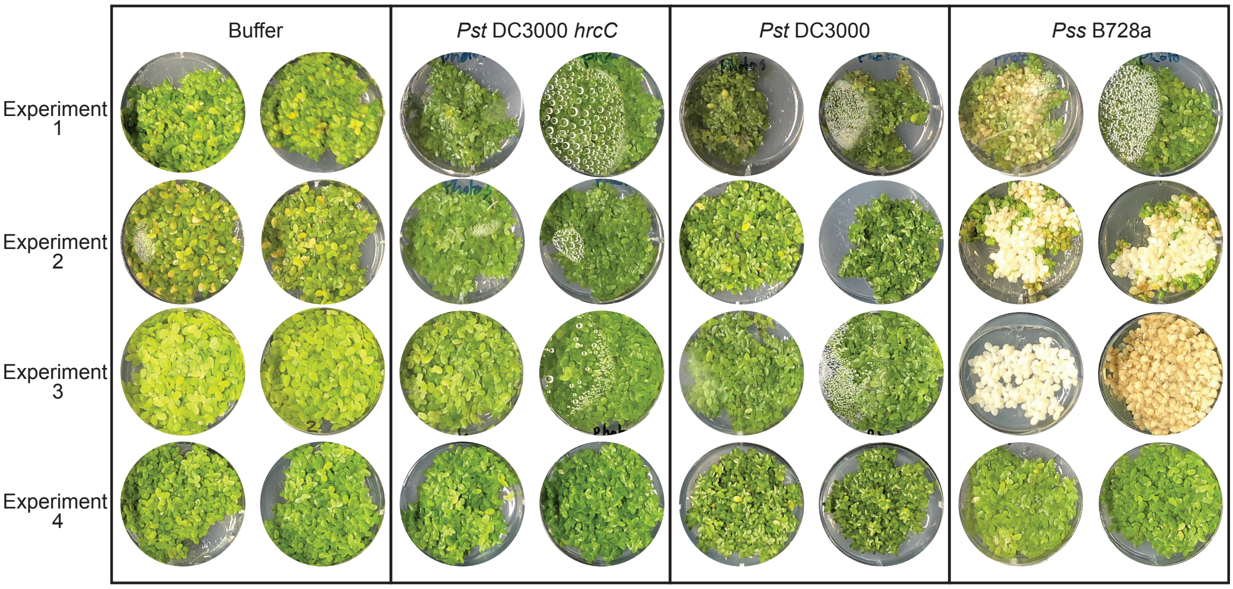

Fig. S12. Low bacterial load infection of *Landoltia punctata* one month post inoculation.
All wells of an experiment started with 12 fronds and were treated on the same day, each well is a separate biological replicate. There are two replicates photographed per treatment. Some wells have condensation which can be seen as the water droplets obscuring fronds below, this is as plates need to be sealed to prevent contamination.

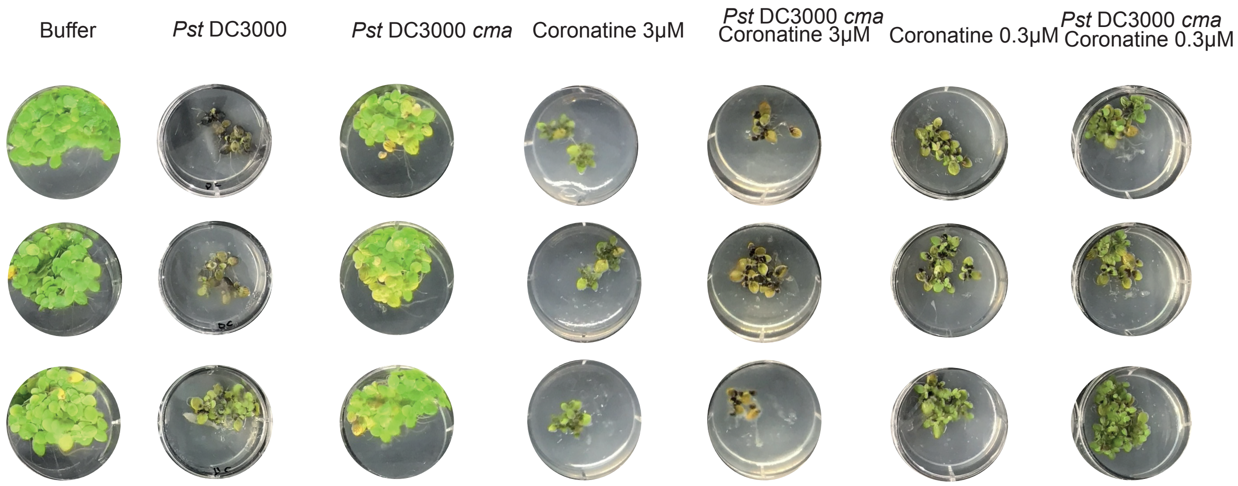

Fig. S13. Role of coronatine in *Pst* DC3000 infection of *Spirodela polyrhiza*.
Experiment 2 of coronatine response experiment Fig, 5a. All wells were treated on the same day and each well is a separate biological replicate.

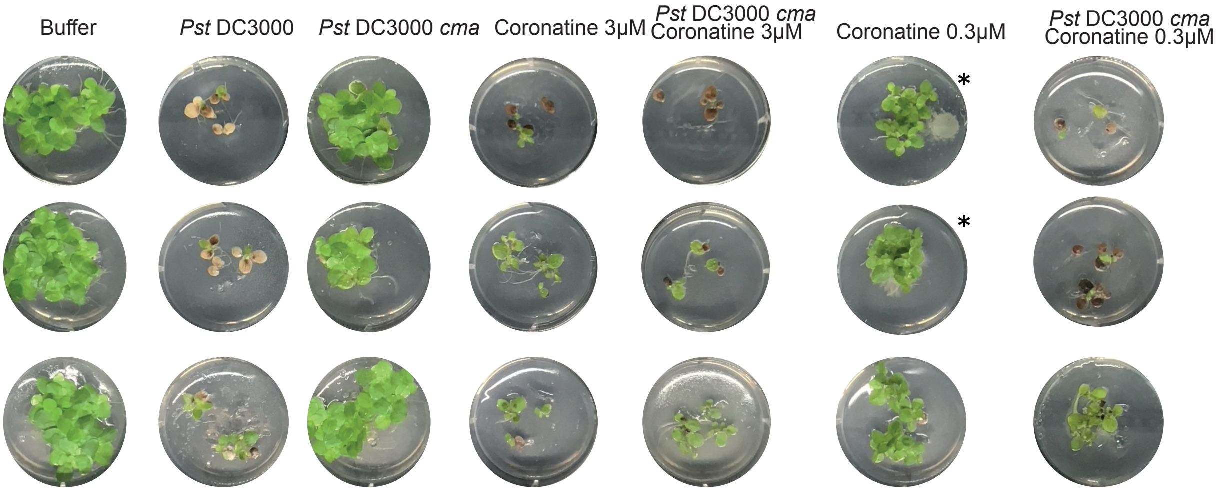

Fig. S14. Role of coronatine in *Pst* DC3000 infection of *Spirodela polyrhiza*.
Experiment 3 of coronatine response experiment Fig. 5a. All wells were treated on the same day and each well is a separate biological replicate. Asterix marks wells with visible contamination.

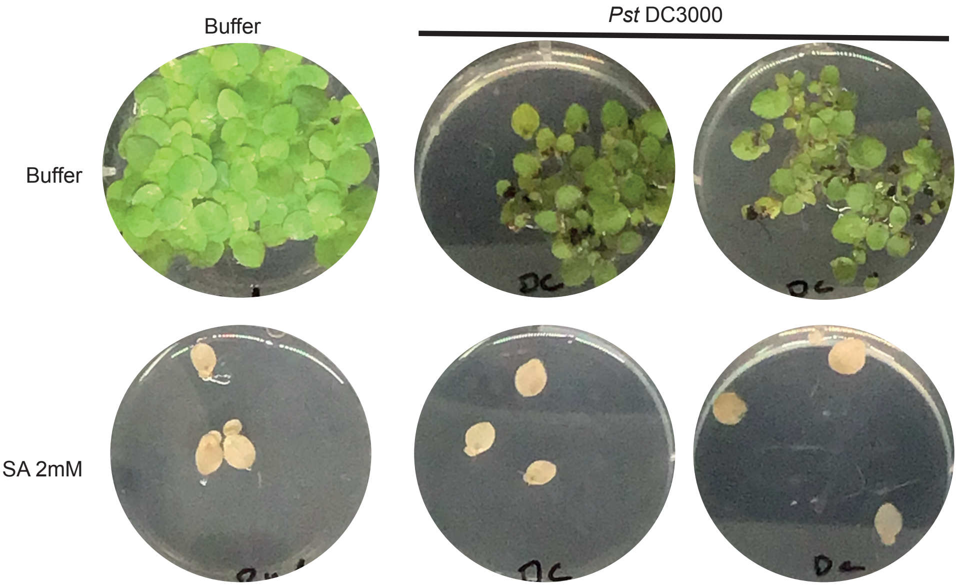

Fig. S15. Salicylic acid phytotoxicity to *Spirodela polyrhiza* upon buffer or *Pst* DC3000 treatment.
Inoculation with SA 24hrs before pathogen inoculation. Images three weeks after treatment with buffer (10mM MgCl2) and *Pst* DC3000.

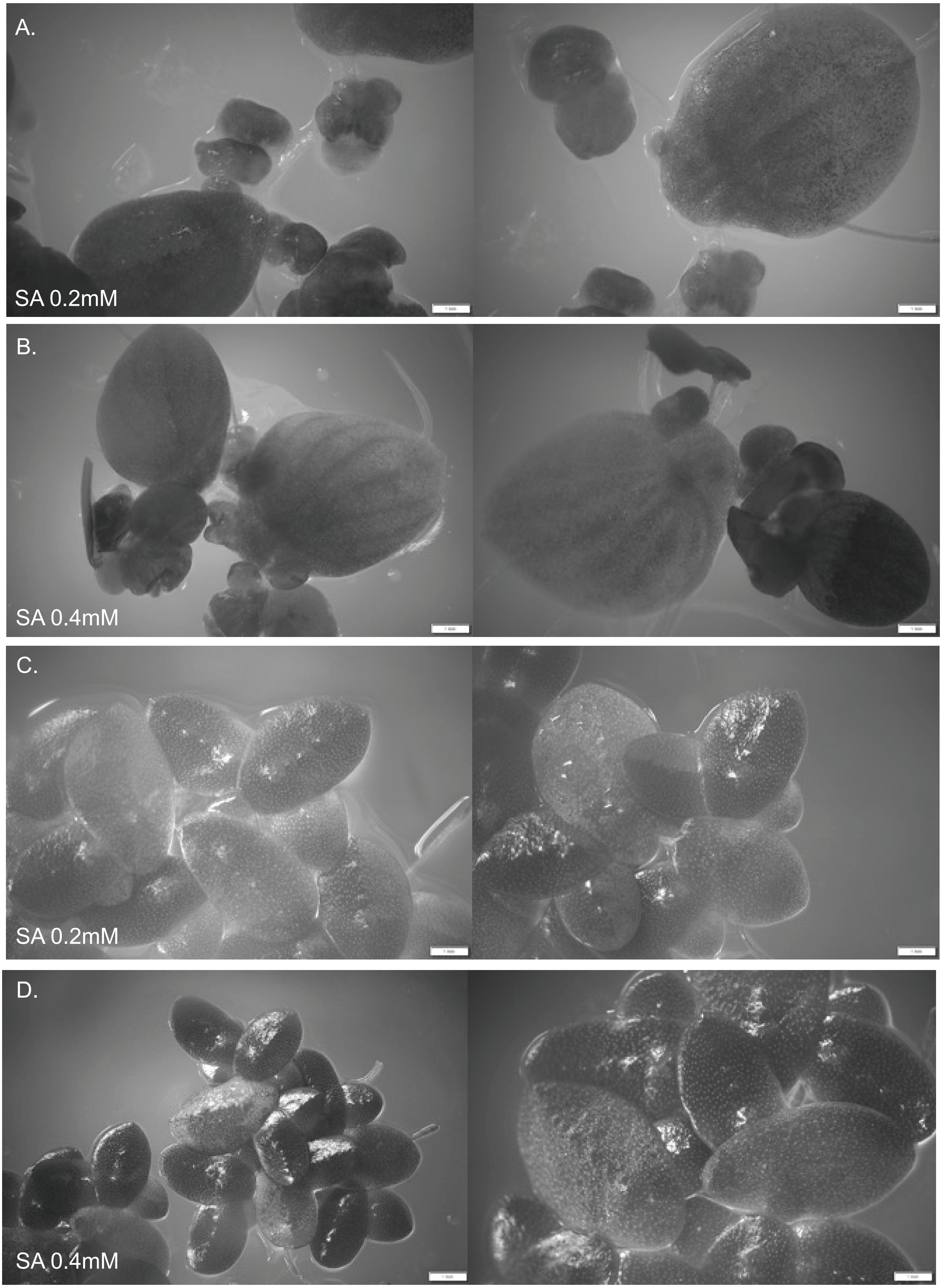

Fig. S16. Dissecting microscope images of duckweed twelve days post inoculation with SA.
A. *S. polyrhiza* mother fronds and turions 12dpi 0.2mM SA. B. *S. polyrhiza* mother fronds and turions 12dpi 0.4mM SA. Frond bleaching is visible from the white frond color. C. *L. punctata* mother and daughter fronds 12dpi 0.2mM SA. D. *L. punctata* mother and daughter fronds and turions 12dpi 0.4mM SA. Scale bars are 1 mm.

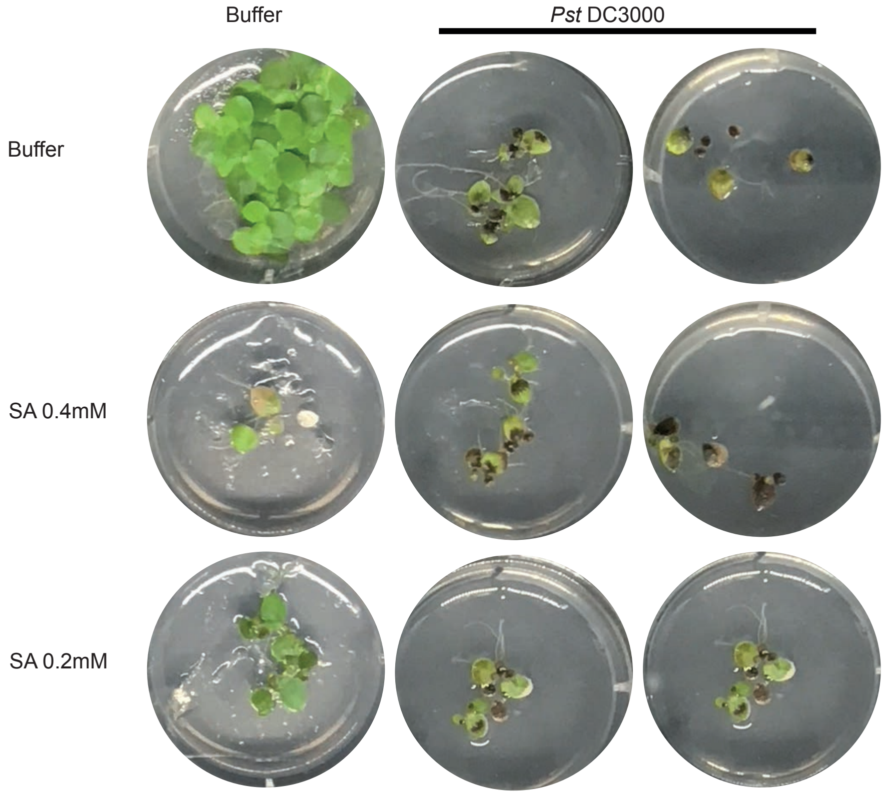

Fig. S17. Role of salicylic acid in *Pst* DC3000 infection of *Spirodela polyrhiza*. Experiment 2 of salicylic acid treatment of *S. polyrhiza* experiment Fig. 5b. All wells were treated on the same day and each well is a separate biological replicate.

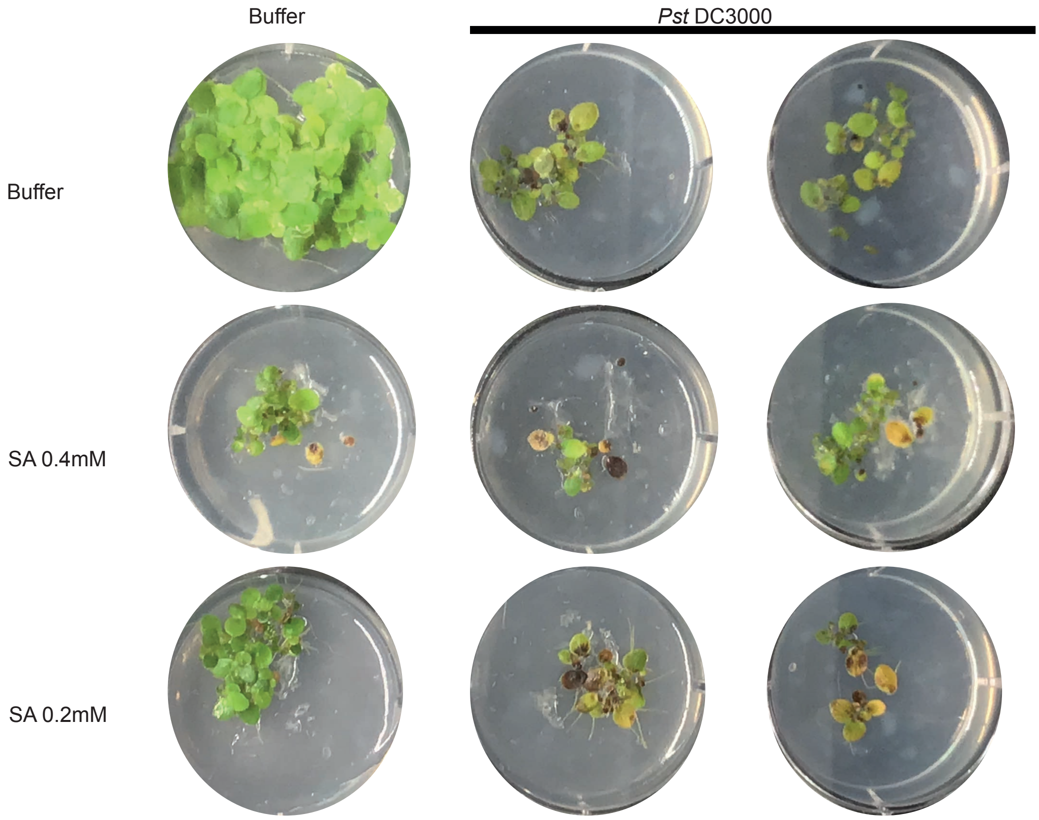

Fig. S18. Role of salicylic acid in *Pst* DC3000 infection of *Spirodela polyrhiza.*
Experiment 3 of salicylic acid treatment of *S. polyrhiza* experiment Fig. 5b. All wells were treated on the same day and each well is a separate biological replicate.

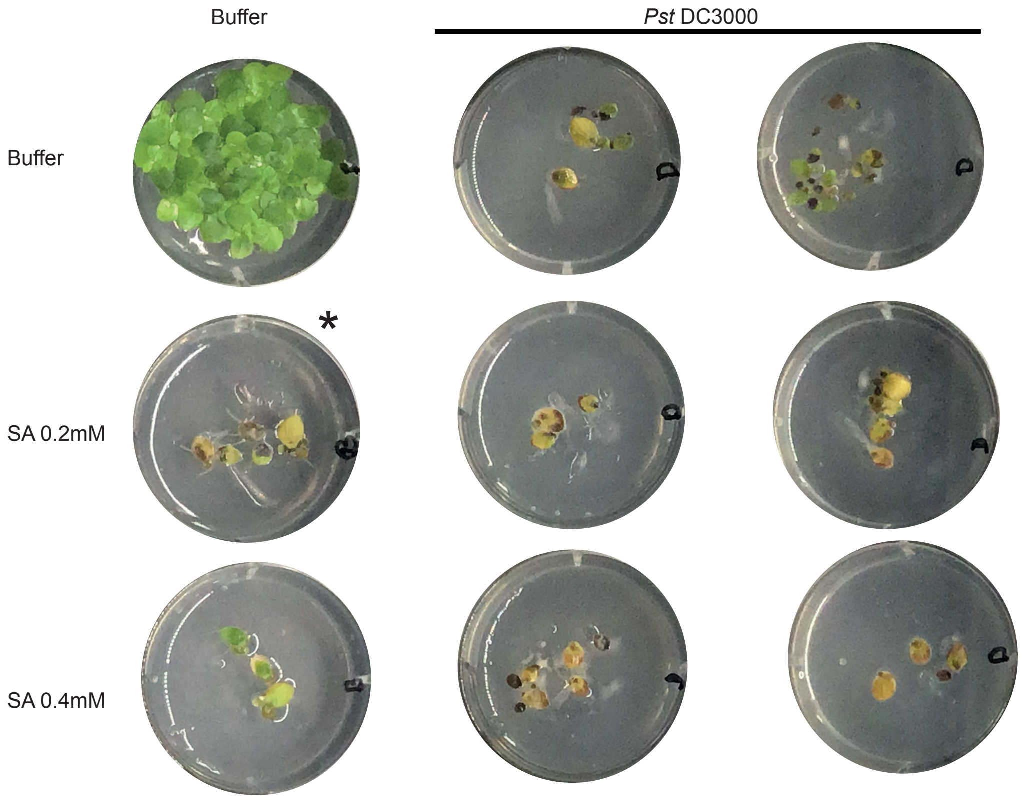

Fig. S19. Role of salicylic acid in *Pst* DC3000 infection of *Spirodela polyrhiza.*
Experiment 4 of salicylic acid treatment of *S. polyrhiza* experiment Fig. 5b. All wells were treated on the same day and each well is a separate biological replicate. Asterisk marks wellswith visible contamination of the buffer with *Pst* DC3000.

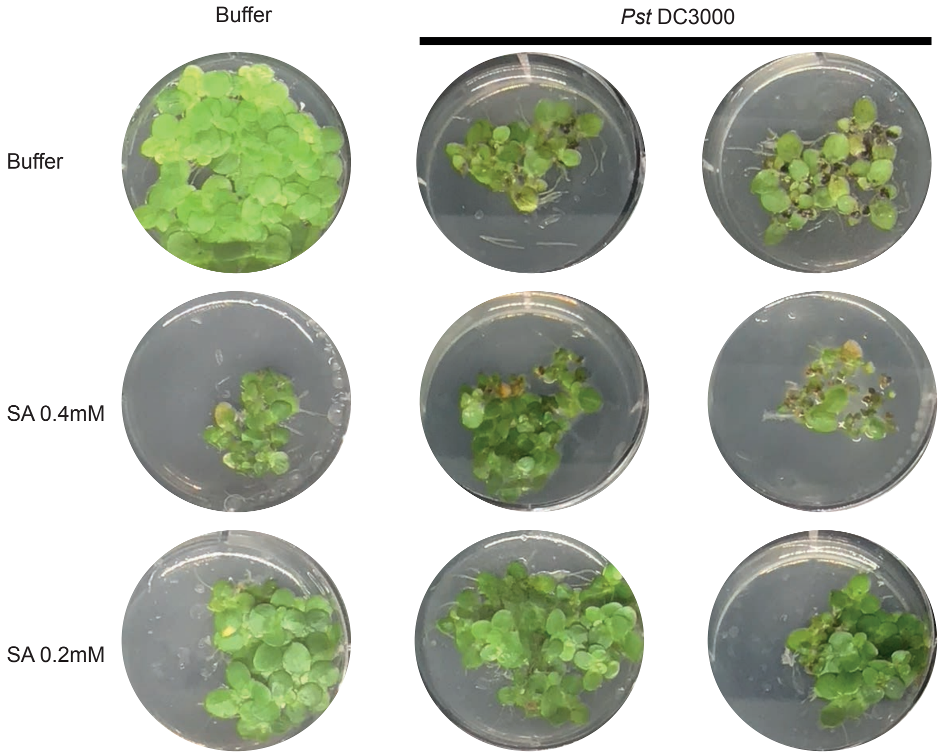

Fig. S20. Role of salicylic acid in *Pst* DC3000 infection of *Spirodela polyrhiza*.
Experiment 5 of salicylic acid treatment of *S. polyrhiza* experiment Fig. 5b. All wells were treated on the same day and each well is a separate biological replicate. Asterisk marks wells with visible contamination of the buffer with *Pst* DC3000.

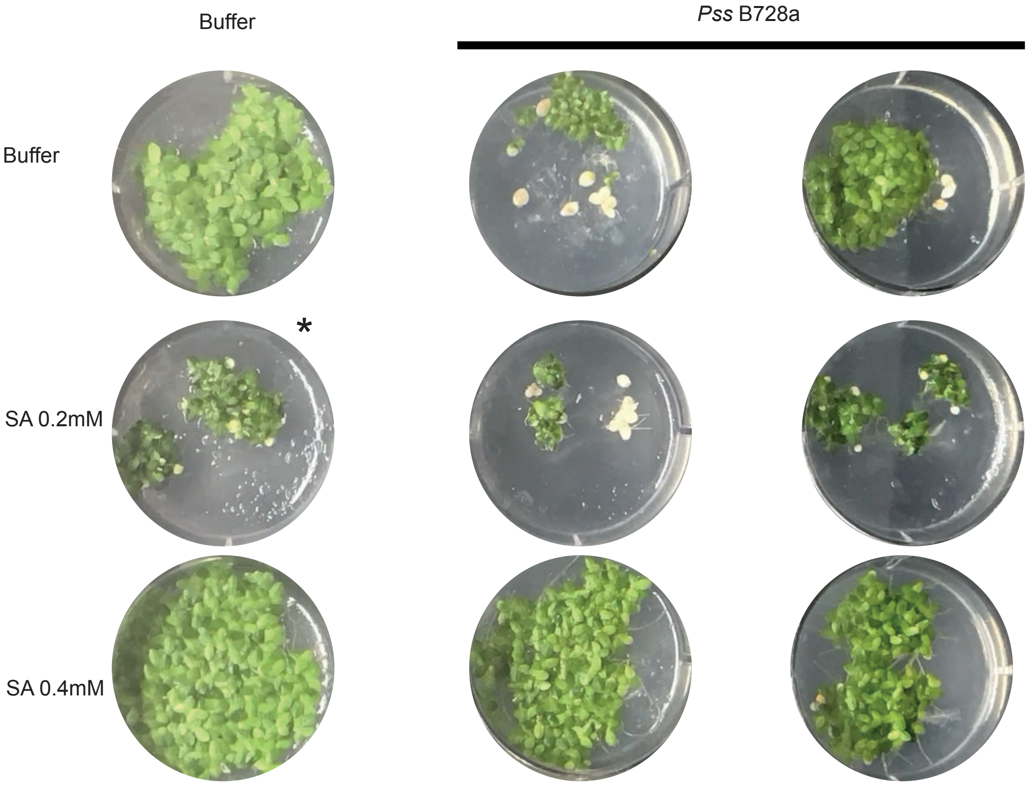

Fig. S21. Role of salicylic acid in *Pss* B728a infection of *Landoltia punctata*.
Experiment 2 of salicylic acid treatment of *L. punctata* experiment Fig. 5c. All wells were treated on the same day and each well is a separate biological replicate. Asterisk marks wells with visible contamination of the buffer with *Pss* B728a.

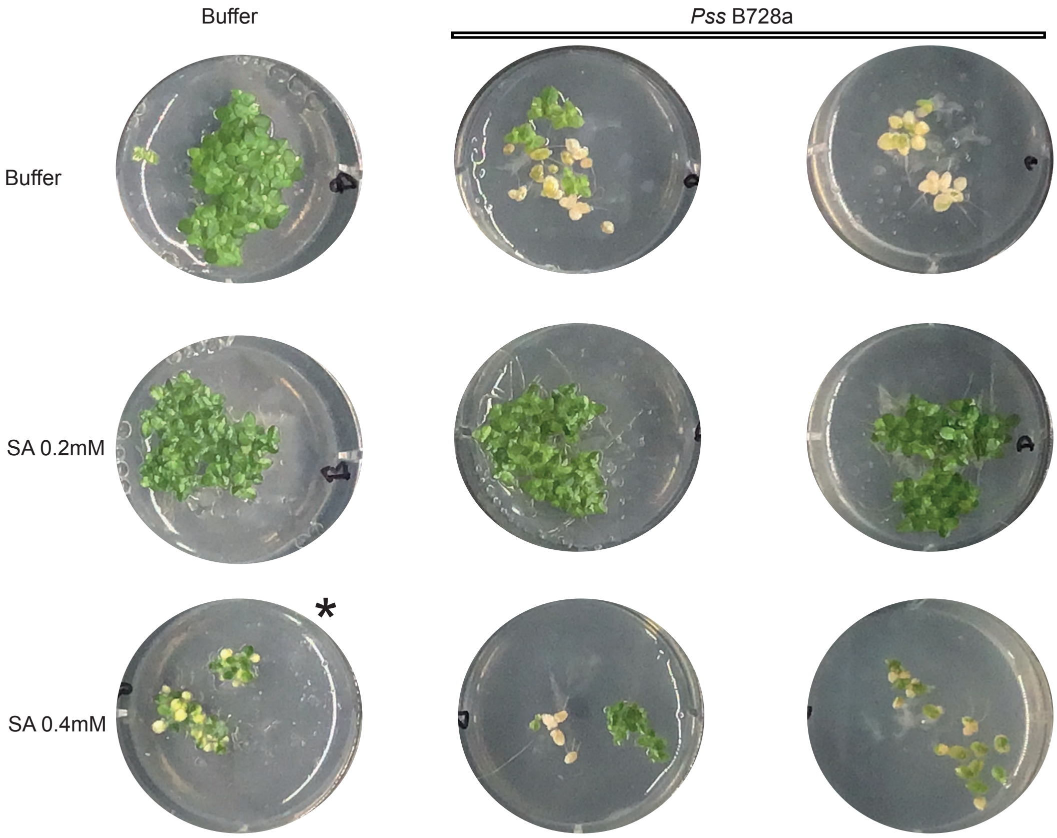

Fig. S22. Role of salicylic acid in *Pss* B728a infection of *Landoltia punctata.*
Experiment 2 of salicylic acid treatment of *L. punctata* experiment Fig. 5c. All wells were treated on the same day and each well is a separate biological replicate. Asterisk marks wells with visible contamination of the buffer with *Pss* B728a.

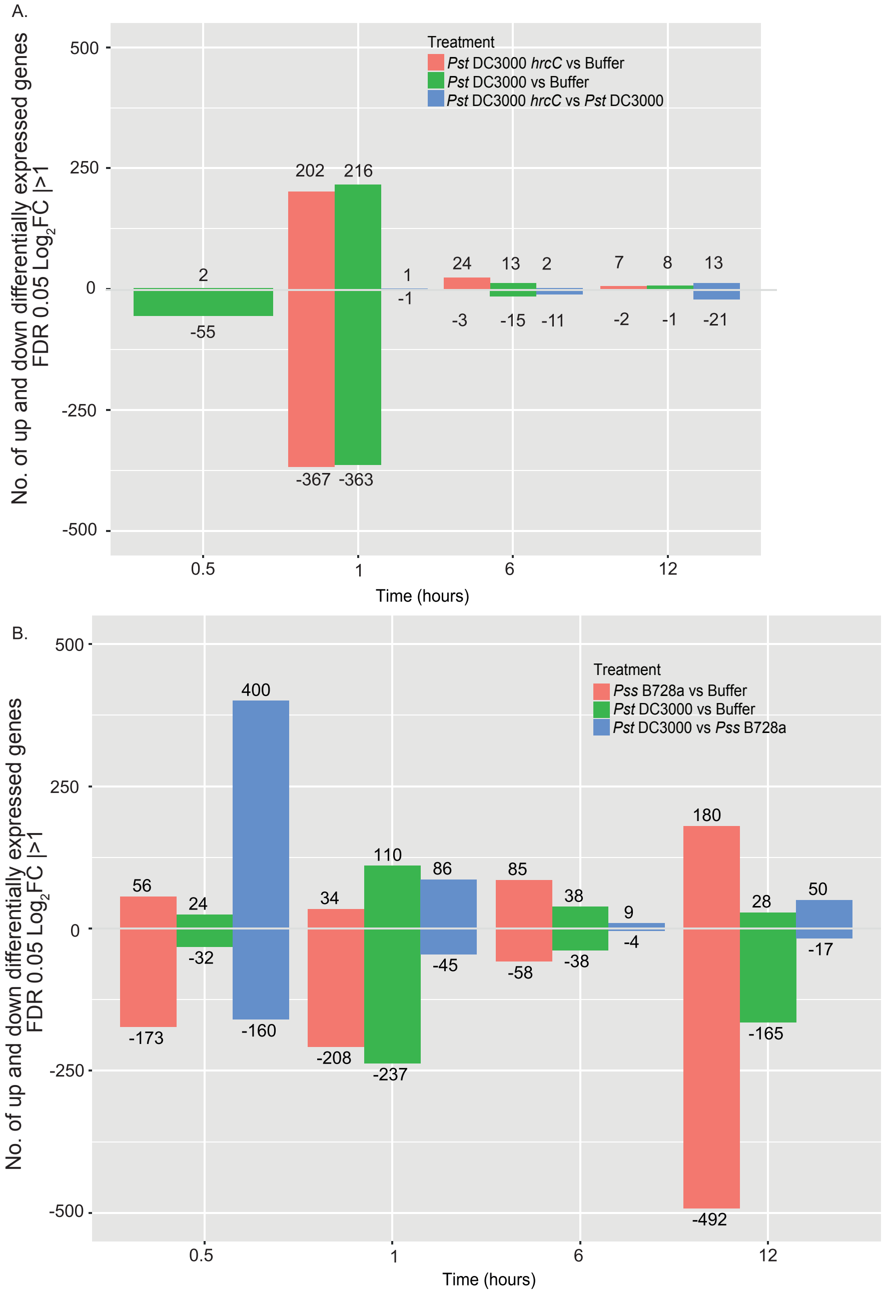

Fig. S23. Barchart of number of genes differentially expressed upon bacterial treatments. Bars indicate no. of up and down differentially expressed genes FDR 0.05 Log_2FC | >1. A. *S. polyrhiza* differentially expressed genes. B. *L. punctata* differentially expressed genes.

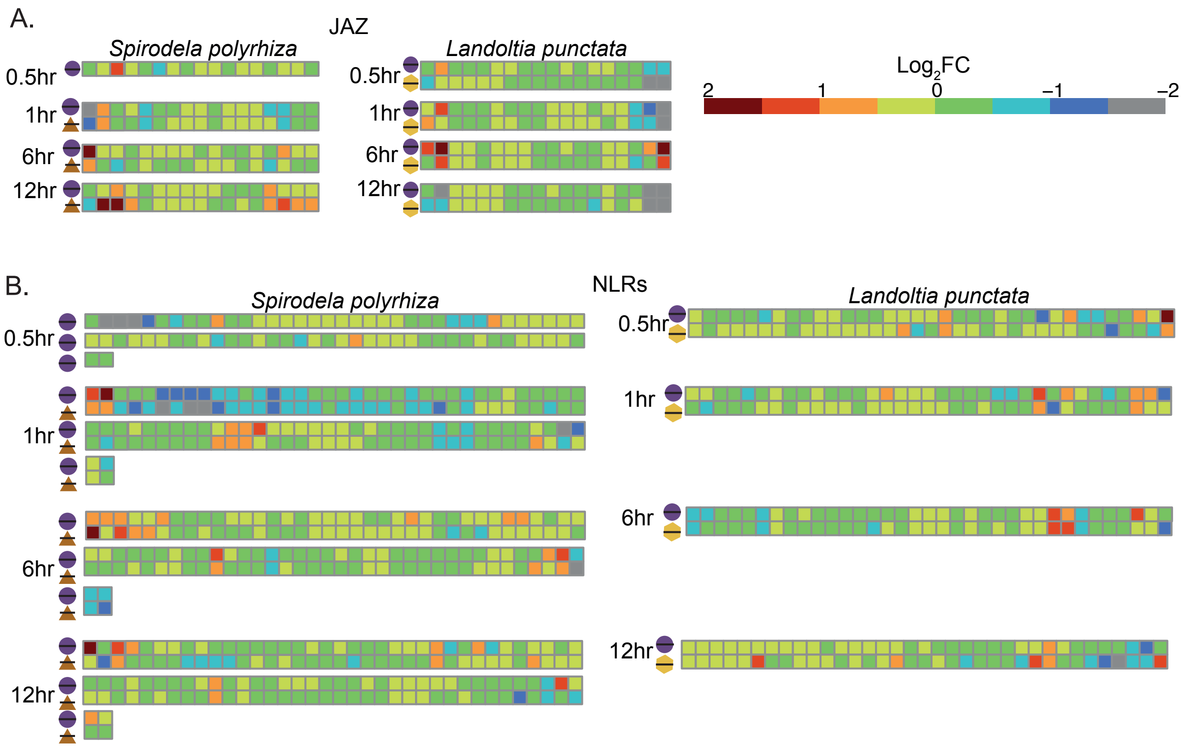

Fig. S24. Log$_2$ fold change of selected gene families following bacterial pathogen exposure of duckweeds. Each square represents a gene with a given domain, the differential expression of the gene is shown by the color of the square. The treatment comparison is indicated by the shapes; black line - buffer, purple circle-*Pst* DC3000, brown triangle -*Pst* DC3000 *hrcC* and yellow hexagon - *Pss* B728a. JAZ domain containing genes are shown in A and NB-ARC containing genes are indicated in B.

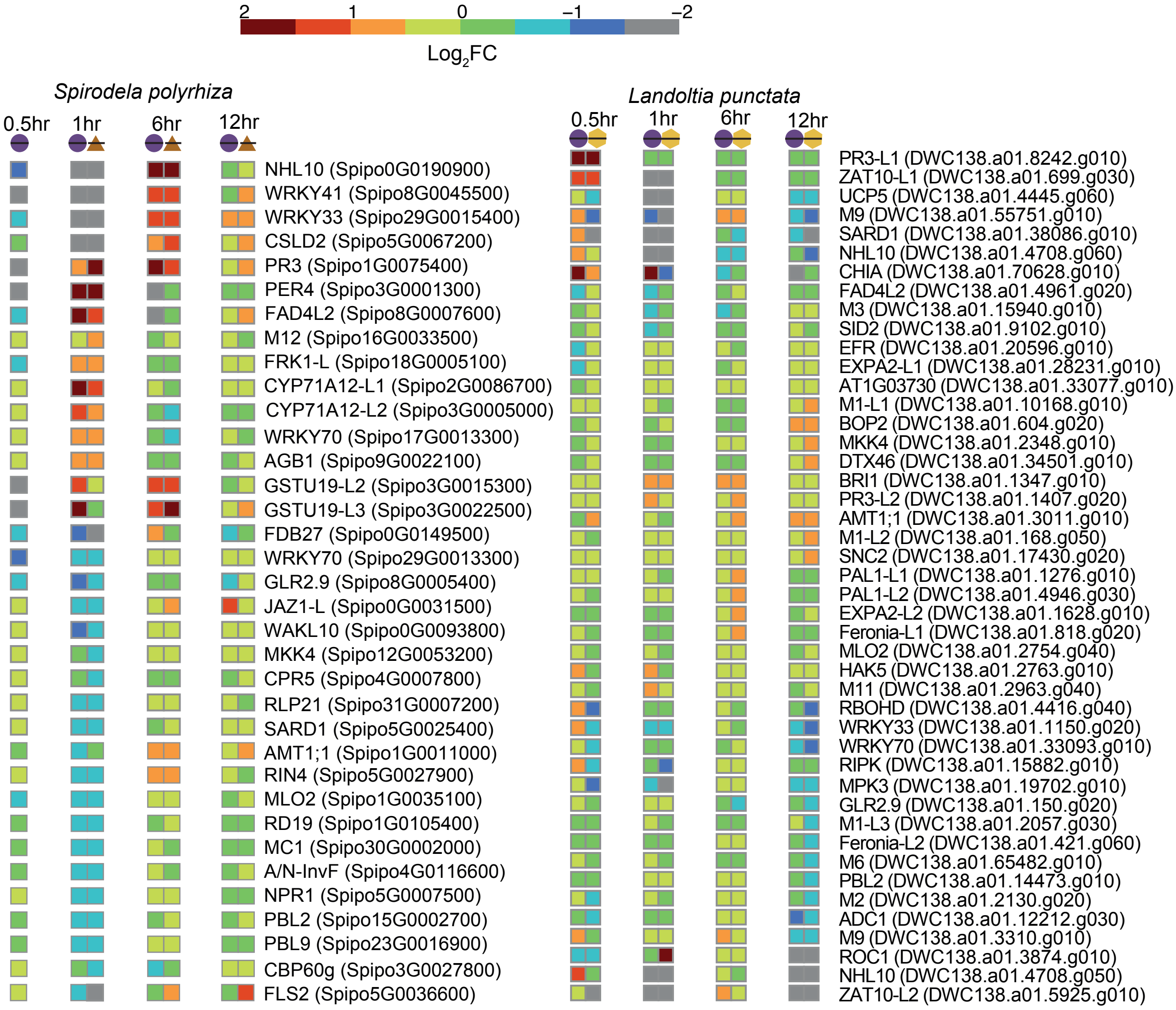

Fig. S25. Log$_2$ fold change of homologs of *A. thaliana* bacterial responsive genes.
Color of square indicates Log$_2$ fold change of homologous genes to *A. thaliana* gene indicated at the end of the row, in brackets is the duckweed gene identified as homologous. The treatment comparison is indicated at the top of the column by the shapes; black line - buffer, purple circle - *Pst* DC3000, brown triangle - *Pst* DC3000 *hrcC* and yellow hexagon - *Pss* B728a.

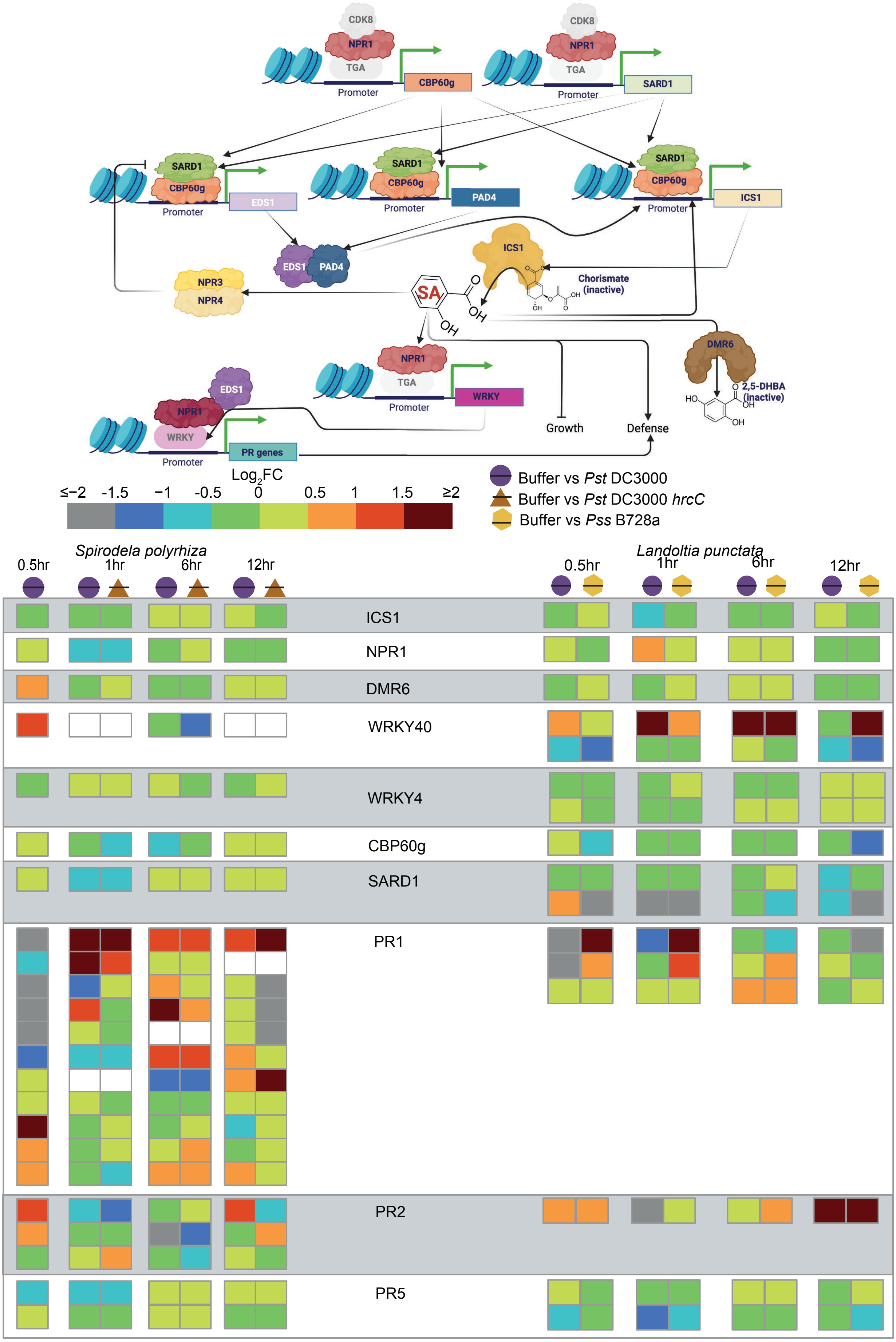

Fig. S26. Differential expression upon pathogen treament of the duckweed homologs of Arabidopsis SA marker genes

A. Schematic of known roles from *A. thaliana* of genes in SA pathway. B. Each line of squares represents a duckweed homolog to the A. thaliana gene named in the same column.The differential expression of the gene is shown by the color of the square. The treatment comparison is indicated by the shapes; black line - buffer, purple circle-*Pst* DC3000, brown triangle - *Pst* DC3000 *hrcC* and yellow hexagon - *Pss* B728a. Gene IDs of homologs to *A. thaliana* marker genes are available in Table S16.

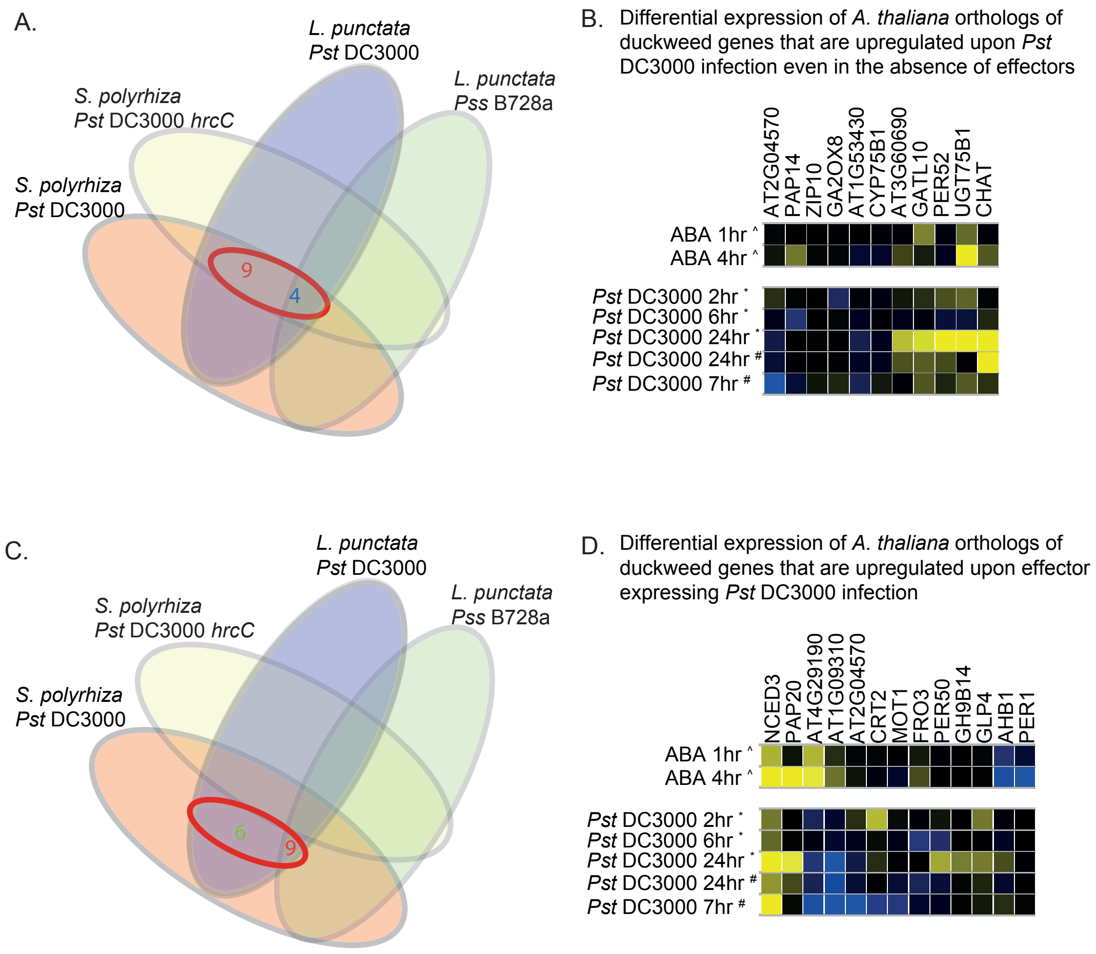

Fig.S27. Orthogroups with conserved upregulation upon pathogen treatment of duckweed species and the expression of orthologs in *A. thaliana.*

A. Venn diagram highlighting numbers of orthogroups that are upregulated (Log_2 FC >1, FDR < 0.05) in *L. punctata* / *Pst* DC3000 and *S. polyrhiza* / *Pst* D3000 and *Pst* DC3000 *hrcC* . B. Microarray differential expression analysis of representatives from *A. thaliana* Col-0 of orthogroups highlighted in A whose upregulation in duckweed is observed in the *Pst* DC3000 *hrcC* mutant suggesting their upregulation is independent of effectors. Source of microarray data is indicated by symbol; ^ Umezawa *et al*. 2010, * Kemmerling *et al.* 2011 and # Thilmony *et al.* 2006. C. Venn diagram highlighting numbers of orthogroups that are upregulated (Log_2 FC >1, FDR < 0.05) in *L. punctata* / *Pst* DC3000 and *S. polyrhiza* / *Pst* D3000 but not in *Pst* DC3000 *hrcC*. D. Microarray expression patterns of subset of genes from C. whose upregulation in duckweed is absent in the *Pst* DC3000 *hrcC* mutant suggesting expression is affected by pathogen effectors. Source of microarray data is indicated as in B.

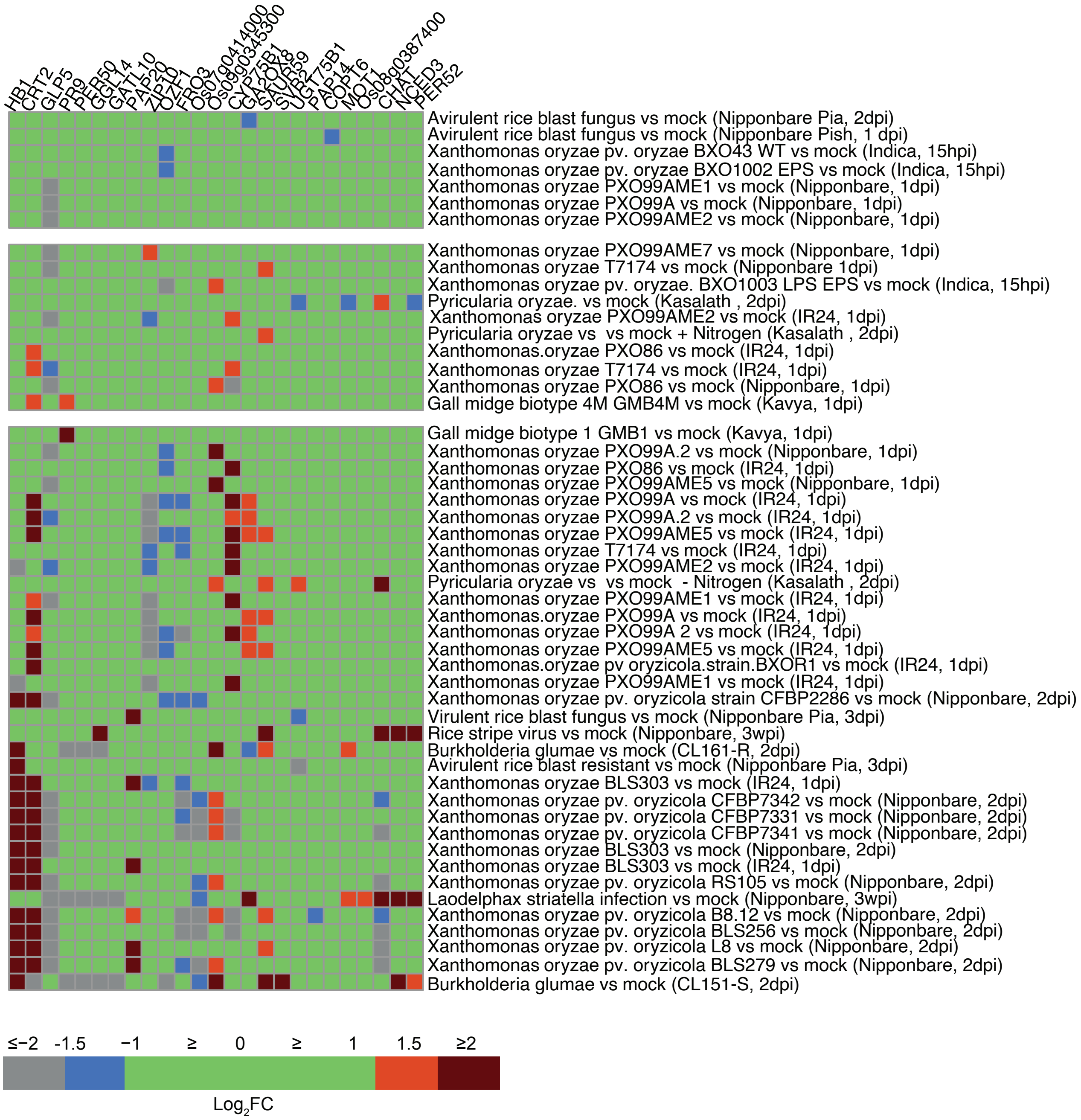

Fig. S28. Heatmap displaying Log_2FC of rice homologs of duckweed conserved upregulated gene upon pathogen infection.

One homolog per orthogroup is shown, condition by gene combinations that resulted in Log_2FC |> 2 and P-value <0.005 are represented by colored squares. Columns are labeled by comparison used to generate the data with brackets showing rice cultivar followed by the timepoint of RNA sampling after inoculation. Data is from the EBI expression atlas studies E-CURD-37, E-GEOD-22538, E-GEOD-36272, E-GEOD-61833, E-GEOD-62894, E-GEOD-67588, E-MTAB-4406, E-MTAB-5025 (Athar et al. 2019; Magbanua et al. 2014; Wilkins et al. 2015; Girija et al. 2017; Huang et al. 2017; Nishimura et al. 2016; Rawat et al. 2012; Zhao et al. 2016).

References

Athar A, Füllgrabe A, George N, Iqbal H, Huerta L, Ali A, Snow C, Fonseca NA, Petryszak R, Papatheodorou I, et al. 2019. ArrayExpress update - from bulk to single-cell expression data. Nucleic acids research 47: D711–D715.

Girija AM, Kinathi BK, Madhavi MB, Ramesh P, Vungarala S, Patel HK, Sonti RV. 2017. Rice Leaf Transcriptional Profiling Suggests a Functional Interplay Between *Xanthomonas oryzae* pv. oryzae Lipopolysaccharide and Extracellular Polysaccharide in Modulation of Defense Responses During Infection. Molecular plant-microbe interactions: MPMI 30: 16–27.

Huang H, Nguyen Thi Thu T, He X, Gravot A, Bernillon S, Ballini E, Morel J-B. 2017. Increase of fungal pathogenicity and role of plant glutamine in nitrogen-Induced Susceptibility (NIS) to rice blast. Frontiers in plant science 8: 265.

Kemmerling B, Halter T, Mazzotta S, Mosher S, Nürnberger T. 2011. A genome-wide survey for Arabidopsis leucine-rich repeat receptor kinases implicated in plant immunity. Frontiers in plant science 2: 88.

Magbanua ZV, Arick M 2nd, Buza T, Hsu C-Y, Showmaker KC, Chouvarine P, Deng P, Peterson DG, Lu S. 2014. Transcriptomic dissection of the rice-*Burkholderia glumae* interaction. BMC genomics 15: 755.

Nishimura T, Mochizuki S, Ishii-Minami N, Fujisawa Y, Kawahara Y, Yoshida Y, Okada K, Ando S, Matsumura H, Terauchi R, et al. 2016. *Magnaporthe oryzae* Glycine-Rich Secretion Protein, Rbf1 Critically Participates in Pathogenicity through the Focal Formation of the Biotrophic Interfacial Complex. PLoS pathogens 12: e1005921.

Rawat N, Naga NC, Meenakshi SR, Nair S, Bentur JS. 2012. A novel mechanism of gall midge resistance in the rice variety Kavya revealed by microarray analysis. Functional & integrative genomics 12: 249–264.

Thilmony R, Underwood W, He SY. 2006. Genome-wide transcriptional analysis of the *Arabidopsis thaliana* interaction with the plant pathogen *Pseudomonas syringae* pv. tomato DC3000 and the human pathogen *Escherichia coli* O157:H7. The Plant journal: for cell and molecular biology 46: 34–53.

Umezawa T, Shinozaki K, Mizoguchi M, Takasaki H, Yamaguchi-Shinozaki K, Kidokoro S, Nakashima K, Fujita Y. 2010. Two Closely Related Subclass II SnRK2 Protein Kinases Cooperatively Regulate Drought-Inducible Gene Expression. Plant & cell physiology 51: 842–847.

Wilkins K, Booher N, Wang L, Bogdanove A. 2015. TAL effectors and activation of predicted host targets distinguish Asian from African strains of the rice pathogen *Xanthomonas oryzae* pv. oryzicola while strict conservation suggests universal importance of five TAL effectors. Frontiers in plant science 6: 536.

Zhao W, Yang P, Kang L, Cui F. 2016. Different pathogenicities of Rice stripe virus from the insect vector and from viruliferous plants. The New phytologist 210: 196–207.
